# Effectiveness of a Multicomponent Intervention to Reduce Multidrug-Resistant Organisms in Nursing Homes: A Cluster Randomized Clinical Trial

**DOI:** 10.1001/jamanetworkopen.2021.16555

**Published:** 2021-07-16

**Authors:** Lona Mody, Kyle J. Gontjes, Marco Cassone, Kristen E. Gibson, Bonnie J. Lansing, Julia Mantey, Mohammed Kabeto, Andrzej Galecki, Lillian Min

**Affiliations:** 1 Division of Geriatric and Palliative Medicine, Department of Internal Medicine, University of Michigan Medical School, Ann Arbor; 2 Geriatrics Research Education and Clinical Center, Veterans Affairs Ann Arbor Healthcare System, Ann Arbor, Michigan; 3 Department of Epidemiology, University of Michigan School of Public Health, Ann Arbor; 4 Department of Biostatistics, University of Michigan School of Public Health, Ann Arbor

## Abstract

**Importance:**

Multidrug-resistant organisms (MDROs) can cause significant morbidity and mortality. Preventing MDROs can reduce the risk of subsequent transmission and infection.

**Objective:**

To determine whether a multicomponent infection prevention intervention can reduce MDRO prevalence in nursing homes (NHs).

**Design, Setting, and Participants:**

This cluster randomized clinical trial of a multicomponent intervention was conducted in 6 NHs in Michigan from September 2016 to August 2018. Three NHs adopted a multicomponent intervention, while 3 control NHs continued without investigator intervention. Study visits were conducted at baseline; days 7, 14, 21, and 30; and monthly thereafter for up to 6 months or discharge. Visits included clinical data collection and MDRO surveillance culturing of multiple body sites and high-touch surfaces in patient rooms. Any patients who provided informed consent within 14 days of admission to the NH were enrolled in this study. Non-English speakers and patients receiving hospice care were ineligible. Analysis was performed from November 2018 to February 2020.

**Interventions:**

Intervention NHs adopted a multicomponent intervention that included enhanced barrier precautions, chlorhexidine bathing, MDRO surveillance, environmental cleaning education and feedback, hand hygiene promotion, and health care worker education and feedback. Control nursing homes continued standard care practices.

**Main Outcomes and Measures:**

The primary outcome, presence of MDROs, was measured longitudinally in the patient and room environment and was evaluated using generalized mixed effect models. The secondary outcome, time to new MDRO acquisition, was assessed using Cox proportional hazard models.

**Results:**

A total of 6 NHs were included, with 245 patients (mean [SD] age, 72.5 [13.6] years; 134 [54.7%] women) enrolled; 3 NHs with 113 patients (46.1%) were randomized to the intervention group and 3 NHs with 132 patients (53.9%) were randomized to the control group. A total of 132 patients (53.9%) were White, and 235 patients (95.9%) were receiving postacute care. Over 808 study visits, 3654 patient cultures and 5606 environmental cultures were obtained. The intervention reduced the odds of MDRO prevalence in patients’ environment by 43% (aOR, 0.57; 95% CI, 0.35-0.94), but there was no statistically significant difference on the patient level before or after adjustment (aOR, 0.57; 95% CI, 0.29-1.14). There were no significant reductions in time to new acquisition for methicillin-resistant *Staphylococcus aureus* (hazard ratio [HR], 0.20; 95% CI, 0.04-1.09), vancomycin-resistant enterococci (HR, 0.84; 95% CI, 0.46-1.53), or resistant gram-negative bacilli (HR, 1.14; 95% CI, 0.73-1.78).

**Conclusions and Relevance:**

This cluster randomized clinical trial found that the multicomponent intervention reduced the prevalence of MDROs in the environment of NH patients. Our findings highlight the potential for multicomponent interventions to directly and indirectly reduce MDRO prevalence in NHs.

**Trial Registration:**

ClinicalTrials.gov Identifier: NCT02909946

## Key Points

**Question:**

What is the effect of a multicomponent infection prevention intervention on multidrug-resistant organisms in nursing homes?

**Findings:**

In this cluster randomized clinical trial of 6 nursing homes, including 245 patients, a multicomponent intervention consisting of enhanced barrier precautions, chlorhexidine bathing, microbial surveillance, and staff engagement statistically significantly reduced the odds of multidrug-resistant organism contamination in patients’ environments.

**Meaning:**

These findings suggest that multicomponent interventions can be tailored to reduce multidrug-resistant organisms burden and transmission potential in nursing homes.

## Teaser

This cluster randomized clinical trial examines the effectiveness of a multicomponent infection prevention intervention for multidrug resistant organisms in nursing homes.

## Introduction

Multidrug-resistant organisms (MDROs) are endemic to nursing homes (NHs), with prevalence rates surpassing those in hospitals.^1^ Patients in NHs with complex, high-acuity conditions are at heightened risk for MDRO colonization, as they often have wounds, recent antibiotic exposures, multiple comorbidities, and indwelling devices.^1,2^ Subsequently, MDRO colonization can lead to increased morbidity and mortality.^1,2,3,4,5^

With MDRO prevalence estimated at greater than 50%,^6,7^ NH patients may serve as reservoirs with important roles in intrafacility transmission. Research in NHs has indicated that environmental reservoirs also play important roles in MDRO transmission.^3,7,8,9,10,11,12,13^ For example, residence in contaminated rooms is associated with an increased risk of MDRO acquisition,^3^ while room environment contamination is associated with patient colonization status.^12,13^

Multicomponent interventions, particularly those based on hand hygiene promotion, staff education, or chlorhexidine gluconate bathing, have successfully reduced MDRO burden in hospitals.^14,15,16,17^ Less is known about the capacity of multicomponent interventions to reduce MDRO patient colonization and room environment contamination in NHs. Therefore, we conducted a cluster randomized clinical trial in 6 Michigan NHs, in a study population consisting predominantly of patients receiving postacute care, to assess whether a multicomponent evidence-based infection prevention intervention would reduce the prevalence of MDROs that colonize patients and contaminate their environment, MDRO acquisition, and the incidence of health care–associated infections. Given the evidence that MDROs are readily transmitted between patients and their proximal environment,^8^ we used generalized mixed-effect models with multivariable logistic regression and multilevel clustering to evaluate the impact of our intervention on MDRO prevalence in both the patient and their room environment.

## Methods

The cluster randomized clinical trial was approved by the institutional review board at the University of Michigan. All participants or their legally authorized representative provided written informed consent. This study followed the Consolidated Standards of Reporting Trials (CONSORT) reporting guideline for reporting cluster randomized trials. The trial protocol is presented in Supplement 1.

### Study Population and Design

We conducted a cluster randomized clinical trial of recently admitted NH patients in Michigan from September 2016 to August 2018. Eligibility criteria included new admission to the NH and enrollment within 14 days and provision of written informed consent by the patient or their legally authorized representative. Exclusion criteria were non–English speaking and receipt of hospice care. Enrolled patients were followed-up for up to 6 months or discharge. The cluster randomized clinical trial design was chosen to avoid potential intervention contamination that occurs when randomizing patients^18,19^ and to avoid the influence of secular trends.^20^

### Intervention

The multicomponent intervention included 6 factors (Table 1). The intervention was developed after a review of previously published studies in both hospital and nursing home settings. Briefly, the multicomponent intervention included enhanced barrier precautions for patients with high risk, chlorhexidine bathing, MDRO surveillance, environmental cleaning education and feedback, hand hygiene promotion, and health care worker (HCW) education and feedback.

**Table 1.** Overview of Multicomponent Intervention

Intervention component	Intervention target	Intervention facilities (multicomponent intervention)	Control facilities (usual care)
Enhanced barrier precautions for high-risk patients	Enrolled patients with high risk	Enhanced barrier precautions (gowns and gloves) for high-risk patients.	Standard infection prevention practices.
Chlorhexidine bathing	Enrolled patients	Chlorhexidine bathing.	No chlorhexidine bathing.
Surveillance: MDRO and any infections	Nursing home	Patient and environment cultures at baseline; days 7, 14, and 30; and then monthly thereafter until 6 mo or discharge; monthly data feedback was provided.	Patient and environment cultures at baseline; days 7, 14, and 30; and then monthly thereafter until 6 mo or discharge; no feedback provided
Environmental cleaning improvement	Nursing home	Educational in-services and checklists for environmental service staff; monthly data feedback was provided to staff.	Standard environmental cleaning practices; no feedback was provided.
Hand hygiene promotion	Enrolled patients and HCWs	Patients hand hygiene education on enrollment; HCWs received hand hygiene education and pocket-size alcohol-based hand sanitizer.	No patient or HCW hand hygiene education; no hand sanitizer was distributed.
HCW education and monthly feedback	Nursing home	Interactive infection prevention education	No education; study team responded to any concerns when asked for advice.

Abbreviations: HCW, health care worker; MDRO, multidrug-resistant organism.

#### Enhanced Barrier Precautions for Patients With High Risk

Standard precautions and HCW hand hygiene were reinforced for the care of all enrolled intervention patients. Patients with a functional disability, indwelling device (feeding tube or indwelling urinary catheter), or presence of open wounds on enrollment were considered to be at a higher risk for MDRO colonization. For these patients, enhanced barrier precautions were recommended when HCWs assisted patients with direct care, including activities of daily living, touching or providing care to indwelling devices, and during wound care. This was encouraged by hanging personal protective equipment caddies on enrolled patient room doors. Intervention NHs received a supply of gowns and hand hygiene products. Control NHs followed their existing infection prevention policies, including using transmission-based precautions for known MDRO infections (eTable 1 in Supplement 2).

#### Chlorhexidine Bathing

Research personnel (K.E.G. and B.J.L.) trained intervention facility HCWs on the use of chlorhexidine at the start of the study. HCWs were instructed to prioritize the daily chlorhexidine bathing of intervention patients with high risk, with the additional suggestion to use chlorhexidine after incontinence and when providing device care and wound care (up to and around dressings). HCWs were instructed to bathe intervention patients with low risk with chlorhexidine wipes twice per week on their regularly scheduled shower days. Signage was placed in study binders at nurses’ stations, the patient’s room, and the patient’s bathroom. Intervention NHs received a supply of chlorhexidine wipes and warmers. Control NHs did not perform chlorhexidine bathing for any patients.

#### MDRO Surveillance

To assess MDRO colonization, specimens were collected from the patient’s dominant hand, nares, oropharynx, groin, and perianal area on study enrollment; days 7, 14, 21, and 30; and monthly thereafter for up to 6 months. Patient enteral feeding tube insertion site, suprapubic catheter site, and wound specimens were collected when present and accessible. To assess patient room contamination, specimens were collected from the following high-touch, representative environmental surfaces: bed controls, bedside table, call button, toilet seat, doorknob, TV remote, and bedrail. Patient equipment specimens collected, depending on availability, included wheelchair, walker, oxygen pump, intravenous pump, and feeding pump.

#### Environmental Cleaning Education and Feedback

At the beginning of the study, research personnel (K.E.G. and B.J.L.) provided training to environmental services personnel, promoting the daily cleaning and disinfection of high-touch surfaces in patient rooms and common areas. Checklists were provided and suggested to serve as daily guides for cleaning. Alongside the review of existing protocols, research personnel provided yearly education to environmental services personnel on MDRO prevention topics, including the role of the environment in infection prevention, the importance of cleaning high-touch surfaces, and common MDROs in the environment. Monthly feedback of patient room environment contamination was reported to environmental services leadership. Control NHs did not receive any environmental services education or feedback.

#### Hand Hygiene Promotion

Hand hygiene education was provided to intervention patients on enrollment. Patients were encouraged to wash their hands throughout the day, including before meals, when entering and exiting their room, when using common areas, before and after touching wounds or devices, and after using the restroom. NH staff were educated on the indications, technique, and duration of hand hygiene and were encouraged to assist patients with handwashing. Stands with mounted hand wipe dispensers were provided for placement in the dining room, rehabilitation gym, and other common areas. Additionally, pocket-sized hand sanitizers were provided to intervention staff.

#### HCW Education and Monthly Feedback

We designed and implemented interactive infection prevention programming that included small-group education, unit-to-unit education, posters, contests, and quizzes. HCW hand hygiene adherence, gown use, and glove use were pragmatically monitored by research personnel, typically once or twice weekly.^21,22^ Infection surveillance pocket cards were provided to nurses and other HCWs.^20^ Routine feedback was provided to HCWs, covering hand hygiene adherence and HCW hand contamination rates, alongside the monthly reporting of MDRO surveillance results, including new acquisitions and new infections.

### Clinical Data Collection

Research personnel reviewed enrolled patient medical records to collect demographic and clinical data. Functional status was measured using the Physical Self-Maintenance Scale, which ranges from 6 (full independence) to 30 (full dependence). The 6 Physical Self-Maintenance Scale categories are bathing, dressing, feeding, ambulation, grooming, and toileting.^23^ Open wounds were defined as having an open chronic wound that required regular dressing changes or a wound vacuum. This included purulent wounds, vascular ulcers, diabetic ulcers, open surgical wounds, or pressure ulcers greater than stage 1. We defined prolonged hospitalization before NH admission as longer than 14 days.^6^ The Charlson Comorbidity Index was used as a cumulative comorbidity measure.^24^ Prior antibiotic exposure was defined as documented receipt of an antibiotic within 30 days before study enrollment.^6^ Clinical infections were objectively defined as the presence of both a clinical note in the patient’s medical record and the prescription of a systemic antibiotic.^22^ We reviewed the Minimum Data Set 3.0 to confirm the dates and destinations of patient discharges.^25^

### Microbiologic Methods

Specimens were collected using sterile swabs in transport media (BactiSwab; Remel) and assessed for MDROs including methicillin-resistant *Staphylococcus aureus* (MRSA), vancomycin-resistant enterococci (VRE), and resistant gram-negative bacilli (R-GNB) in our research laboratory using previously described, standard microbiologic methods and appropriate quality controls.^6^ We classified gram-negative bacilli as R-GNB if they were resistant to any of the following antibiotics: ceftazidime (30 μg), ceftazidime and clavulanic acid (30/10 μg), ciprofloxacin (5 μg), or imipenem (10 μg).^6^

### Sample Size

Our sample included 6 privately-owned NHs with an mean (range) bed size of 107 (74-143) beds (eTable 2 in Supplement 2). Power calculations were based on the assumption of a 30% reduction in new MDRO acquisition as a result of our intervention. The desired statistical power was 0.80, and significance was set at 2-sided *P* = .05. We adjusted for intracluster correlation using an intraclass correlation coefficient of 0.07.^22^ We estimated that we would need to recruit approximately 239 patients over 2 years in both groups. Assuming a 50% consent rate, we anticipated screening approximately 478 patients to achieve our sample size. Thus, 3 NHs per study arm would suffice in reaching 80% power for our primary aim of reducing MDRO prevalence between the study arms.

### Randomization and Masking

As randomizing participants or HCWs within NHs may lead to the transient crossover of intervention knowledge between subgroups and attenuation of the true effect of the intervention, we cluster randomized NHs. Facilities were randomized by their projected number of recruited participants using computer-generated randomization procedures by the study statisticians (M.K. and A.G.).^22^ While field staff (K.E.G. and B.J.L.) were aware of facility intervention status, the researcher processing microbiological specimens (M.C.) was masked.

### Main Outcomes

The primary study outcome was the presence of patient MDRO colonization or contamination of the patient’s room environment over the duration of the study. The secondary outcome was the new acquisition of MDROs in patients. We also report an exploratory outcome, NH onset of clinically documented infections.

### Statistical Analysis

To evaluate the impact of the intervention on the prevalence of any MDRO and each MDRO (MRSA, VRE, and R-GNB) for patient and environmental specimens separately, we used logistic regression with random effect, a type of generalized mixed-effect model. We performed unadjusted and adjusted analyses (adjusting for age, sex, race/ethnicity, functional status, and device use).

In addition to separate analyses, we performed a post hoc analysis of pooled patient and room environment data and analyzed them jointly to assess the effect of the intervention on MDRO presence in the patient-environment setting (eFigure 1 in Supplement 2). Our analyses included 245 patient-environment dyads, encompassing 1586 total visits. We adjusted for multilevel data by considering 2 random effects: dyads clustered within visits and patient vs environment within each dyad.

To estimate the impact of the intervention on MDRO acquisition, we analyzed data from patients with more than 1 visit who did not have any MDRO of interest at study enrollment. We used univariable and multivariable Cox regression modeling to estimate the time to new acquisition of each MDRO and a combined any new MDRO outcome. The multivariable Cox regression model adjusted for age, sex, race/ethnicity, functional status, and device use. Race/ethnicity was collected via health record review per National Institutes of Health requirements. In an exploratory analysis, we added patient room contamination as an additional risk factor for patient MDRO acquisition to the multivariable Cox regression models. The acquisition rate was defined as new acquisition events per 1000 patient-days. All regression analyses were clustered by NH. Although not powered to test for the intervention’s effect on clinically documented infections, we compared infection rates in each group using Fisher exact test.

Data were analyzed using Stata statistical software version 15.1 (StataCorp). Preliminary analysis was performed from November 2018 to April 2019. Analysis for first submission was performed from October 2019 to February 2020.

## Results

This cluster randomized clinical trial was conducted at 6 NHs, with 3 randomized to the intervention and 3 randomized to the control. Across the 6 NHs, we screened 1491 patients for eligibility, of whom 799 (53.6%) were eligible. Of 799 eligible patients, 245 (30.7%) were enrolled in the study (Figure). In total, 113 patients (46.1%) were enrolled from intervention NHs and followed for 348 study visits (mean [SD], 3.1 [1.9] visits each), and 132 patients (53.9%) were enrolled in control NHs and followed for 460 study visits (mean [SD], 3.5 [2.2] visits each).

**Figure.**
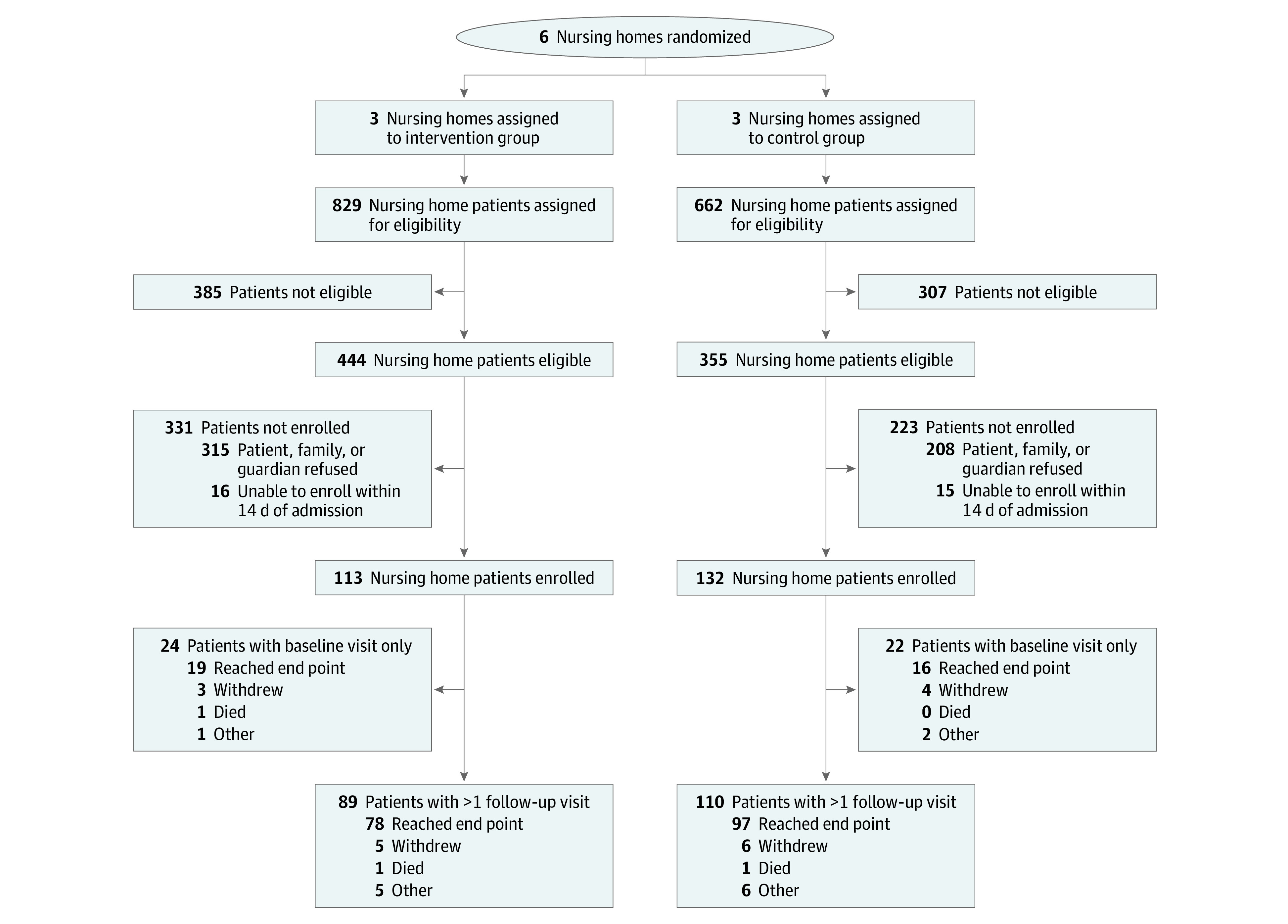
Participant Enrollment Flowchart Patients were enrolled within 14 days of arrival at the nursing home.

### Study Group Characteristics

The mean (SD) age of all enrolled patients was 72.5 (13.6) years, 134 (54.7%) were women, and 132 patients (53.8%) were non-Hispanic White (Table 2). Nearly all patients (231 patients [94.3%]) were admitted for an anticipated short stay, and 235 patients (95.9%) were recorded as receiving postacute care. Patient clinical characteristics were generally balanced between the study arms. However, the intervention NHs had a lower proportion of non-Hispanic White patients than the control NHs (49 patients [43.4%] vs 83 patients [63.4%]). In the acquisition analysis sample, which was restricted to patients with at least 2 study visits, we also found that intervention patients had lower levels of functional disability than the control patients (eTable 3 in Supplement 2). We distributed 11 808 hand sanitizer bottles and 84 450 chlorhexidine wipes to the intervention facilities.

**Table 2.** Characteristics of Patients Receiving Postacute Care on Enrollment

Characteristic	No. (%)^a^	
	Intervention (n = 113)	Control (n = 132)
Age, mean (SD), y	71.6 (13.6)	73.2 (13.6)
Sex		
Men	48 (42.5)	63 (47.7)
Women	65 (57.5)	69 (52.3)
Race		
Non-Hispanic White	49 (43.4)	83 (63.4)
African American	64 (56.6)	48 (36.6)
Antibiotic use in past 30 d	63 (58.3)	68 (54.8)
Charlson Comorbidity Index score, median (IQR)	2 (1-3)	2 (1-4)
Physical Self-Maintenance Scale score, median (IQR)^b^	12 (10.0-15.5)	13.5 (10.0-17.0)
Ambulation	49 (43.4)	71 (54.2)
Dressing	47 (41.6)	78 (59.5)
Bathing	45 (39.8)	69 (52.3)
Toileting	30 (26.6)	46 (35.1)
Grooming	23 (20.4)	39 (29.6)
Feeding	3 (2.7)	7 (5.3)
Device use on enrollment	10 (8.9)	19 (14.4)
Feeding tube	2 (1.8)	5 (3.8)
Urinary catheter	8 (7.1)	15 (11.4)
Percutaneously inserted central line	6 (5.3)	7 (5.3)
History of MDRO		
MRSA	4 (3.7)	3 (2.5)
VRE	1 (0.9)	1 (0.8)
R-GNB	2 (1.9)	4 (3.3)
Open wounds on enrollment	15 (13.5)	19 (15.0)
Admitted from hospital	109 (99.1)	126 (98.4)
Length of preadmission hospitalization >14 d	13 (11.6)	10 (7.6)
Anticipated short stay	108 (96.4)	123 (93.9)
Follow-up, median (IQR), d	14 (6-22)	14 (7-27)

Abbreviations: IQR, interquartile range; MDRO, multidrug-resistant organism; MRSA, methicillin-resistant *Staphylococcus aureus*; R-GNB, resistant gram-negative bacilli; VRE, vancomycin-resistant enterococci.

^a^ Owing to data missing on admission sample sizes were race/ethnicity, 244 patients; antibiotic use in past 30 days, 232 patients; physical self-maintenance scale score, 242 patients; ambulation, 244 patients; dressing, 244 patients; toileting, 244 patients; feeding, 244 patients; history of MRSA, 227 patients; history of VRE, 229 patients; history of R-GNB, 225 patients; open wounds on enrollment, 238 patients; admitted from hospital, 238 patients; length of preadmission hospitalization longer than 14 days, 244 patients; and anticipated short-stay patient, 243 patients.

^b^ Functional disabilities are defined as ambulation: ambulates with assistance of another person, uses a wheelchair with help getting in and out, or cannot move without help; dressing: requires at least moderate assistance with dressing; bathing: unable to independently bathe more than hands and face; toileting: soiling or wetting more than once a week; grooming: regularly needs at least moderate assistance or supervision in grooming; and feeding: eats with moderate assistance and is untidy.

### MDRO Prevalence

We collected 1556 patient specimens from intervention NH patients, of which 245 specimens (15.8%) had test results positive for 1 or more MDROs: 93 specimens (6.0%) with MRSA, 102 specimens (6.6%) with VRE, and 81 specimens (5.2%) with R-GNB (eTable 4 in Supplement 2). We collected 2098 control NH patient specimens, of which 434 specimens (20.7%) had test results positive for 1 or more MDROs: 169 specimens (8.1%) with MRSA, 208 specimens (9.9%) with VRE, and 151 specimens (7.2%) with R-GNB. By univariate analyses, the multicomponent intervention reduced the odds of the presence of any MDRO (odds ratio [OR], 0.72; 95% CI, 0.60-0.85), MRSA (OR, 0.73; 95% CI, 0.56-0.94), VRE (OR, 0.64; 95% CI, 0.50-0.82), and R-GNB (OR, 0.71; 95% CI, 0.54-0.94) in the patient.

We collected 2372 environmental specimens from rooms of patients in the intervention NHs. Of these, 309 specimens (13.0%) had test results positive for 1 or more MDRO: 122 specimens (5.2%) with MRSA, 152 specimens (6.4%) with VRE, and 66 specimens (2.8%) with R-GNB. We collected 3234 environmental specimens from rooms of control NH patients. Of these, 555 specimens (17.2%) had test results positive for 1 or more MDRO: 232 specimens (7.2%) with MRSA, 308 specimens (9.5%) with VRE, and 106 specimens (3.3%) with R-GNB. By univariate analyses, the multicomponent intervention reduced the odds of the presence of any MDRO in the environment (OR, 0.72; 95% CI, 0.62-0.84) and specifically of MRSA (OR, 0.70; 95% CI, 0.56-0.88) and VRE (OR, 0.65; 95% CI, 0.53-0.80) environmental contamination. There was no significant reduction in R-GNB environmental contamination (OR, 0.84; 95% CI, 0.62-1.15). MDRO surveillance data by study visit are presented in eFigure 2 in Supplement 2. In-depth, swab-level MDRO data are presented in eTable 5 and eTable 6 in Supplement 2.

For the primary outcome, after adjusting for patient-level covariates (ie, age, sex, race/ethnicity, functional status, and device use), there was no statistically significant difference between intervention and control patients in overall patient MDRO colonization (adjusted OR, 0.57; 95% CI, 0.29-1.14; *P* = .12). There was a statistically significant reduction in overall environmental MDRO contamination for intervention NHs (adjusted OR, 0.57; 95% CI, 0.35-0.94, *P* = .03) (Table 3).

**Table 3.** Effect of Multicomponent Intervention on MDRO Outcomes

Analysis	OR (95% CI)			
	Any MDRO	MRSA	VRE	R-GNB
**Separate analysis for patient colonization and environment contamination**				
Unadjusted model				
Patient colonization	0.51 (0.25-1.05)	0.59 (0.09-3.78)	0.51 (0.22-1.21)	0.67 (0.35-1.26)
Environment contamination	0.59 (0.35-0.98)^a^	0.57 (0.28-1.16)	0.49 (0.25-0.95)^a^	0.74 (0.44-1.24)
Adjusted model^b^				
Patient colonization	0.57 (0.29-1.14)	0.61 (0.09-4.32)	0.62 (0.28-1.38)	0.71 (0.37-1.37)
Environment contamination	0.57 (0.35-0.94)^a^	0.62 (0.30-1.29)	0.50 (0.26-0.98)^a^	0.63 (0.37-1.07)
**Dyadic analysis for the pooled patient colonization and environment contamination**^**c**^				
Unadjusted model	0.56 (0.33-0.97)^a^	0.39 (0.14-1.06)	0.49 (0.25-0.95)^a^	0.70 (0.44-1.14)
Adjusted model^b^	0.58 (0.35-0.98)^a^	0.44 (0.16-1.20)	0.53 (0.27-1.02)	0.67 (0.41-1.09)

Abbreviations: MDRO, multidrug-resistant organism; MRSA, methicillin-resistant *Staphylococcus aureus*; OR, odds ratio; R-GNB, resistant Gram-negative bacilli; VRE, vancomycin-resistant enterococci.

^a^ *P* < .05.

^b^ Adjusted for age, sex, race/ethnicity, functional status, and device use.

^c^ Adjusted for multilevel data by considering 2 random effects (dyads clustered within visits and patient vs environment within each dyad). Analyses included 245 dyads with 1586 total visits.

When we used multilevel modeling to evaluate the intervention’s effect on the MDRO prevalence in the pooled samples from both the patient and their room environment, also controlling for age, sex, race/ethnicity, functional status, and device usage, we detected a statistically significant 42% reduction in overall MDRO prevalence in the intervention NH patients when compared with control NH patients and their environment (adjusted OR, 0.58; 95% CI, 0.35-0.98; *P* = .04). Among specific MDROs, we found no significant reductions in prevalence of MRSA (adjusted OR, 0.44; 95% CI, 0.16-1.20; *P* = .11), VRE (adjusted OR, 0.53; 95% CI, 0.27-1.02; *P* = .06), or R-GNB (adjusted OR, 0.67, 95% CI, 0.41-1.09; *P* = .11).

### New Acquisitions of MDROs

The rates for a new MDRO acquisition were 36.3 (95% CI 27.2-48.3) infections per 1000 patient-days in the intervention NHs and 42.7 (95% CI 33.6-54.3) acquisitions per 1000 patient-days in the control NHs (Table 4). The rates of new MRSA acquisition were 2.4 (95% CI 0.9-6.4) acquisitions per 1000 patient-days in intervention NHs and 8.0 (95% CI 5.0-12.8) acquisitions per 1000 patient-days in control NHs. The rates of new VRE acquisition were 8.4 (95% CI 4.9-14.4) acquisitions per 1000 patient-days in intervention NHs and 9.0 (95% CI 5.6-14.4) acquisitions per 1000 patient-days in control NH patients. The rates of new R-GNB acquisition were 13.5 (95% CI 8.8-20.7) acquisitions per 1000 patient-days in intervention NHs and 13.9 (95% CI 9.5-20.2) acquisitions per 1000 patient-days in control NHs.

**Table 4.** Effect of Multicomponent Intervention on New MDRO Acquisition Rates

Organism	Intervention group			Control group			Patient covariate–adjusted		Patient- and environment-level adjusted	
	New acquisitions, No.	Follow-up, patient-days	New acquisition rate per 1000 patient-days (95% CI)^a^	New acquisitions, No.	Follow-up, patient-days	New acquisition rate per 1000 patient-days (95% CI)^a^	HR (95% CI)^b^	*P* value	HR (95% CI)^c^	*P* value
Any MDRO (n = 195 patients)	47	1296	36.3 (27.2-48.3)	67	1568	42.7 (33.6-54.3)	0.91 (0.66-1.27)	.59	0.99 (0.66-1.48)	.97
MRSA (n = 168 patients)	4	1667	2.4 (0.9-6.4)	17	2129	8.0 (5.0-12.8)	0.20 (0.04-1.09)	.06	0.19 (0.04-1.07)	.06
VRE (n = 148 patients)	13	1553	8.4 (4.9-14.4)	17	1898	9.0 (5.6-14.4)	0.84 (0.46-1.53)	.57	0.90 (0.49-1.64)	.73
R-GNB (n = 163 patients)	21	1558	13.5 (8.8-20.7)	27	1945	13.9 (9.5-20.2)	1.14 (0.73-1.78)	.57	1.17 (0.73-1.85)	.52

Abbreviations: HR, hazard ratio; MDRO, multidrug-resistant organism; MRSA, methicillin-resistant *Staphylococcus aureus*; R-GNB, resistant gram-negative bacilli; VRE, vancomycin-resistant enterococci.

^a^ 95% CIs were calculated using a quadratic approximation to the Poisson log-likelihood for the log rate estimate.

^b^ Multivariable Cox regression model was adjusted for age, sex, race/ethnicity, functional status, and device use.

^c^ The patient- and environment-level adjusted model added visit-level patient room environment contamination to the multivariable Cox regression model to control for the influence of the room environment on MDRO acquisition.

When adjusting for patient-level covariates, the intervention was not associated with significantly decreased time to new MRSA acquisition (hazard ratio [HR], 0.20; 95% CI, 0.04-1.09; *P* = .06), VRE acquisition (HR, 0.84; 95% CI, 0.46-1.53; *P* = .57), or R-GNB acquisition (HR, 1.14; 95% CI, 0.73-1.78; *P* = .57). These observations were conserved after the addition of patient room environment contamination to the multivariable Cox proportional hazard model.

### Other Adverse Events

We identified 36 new clinically documented infections with onset after admission (eTable 7 in Supplement 2). The most common infections included urinary tract infections (12 infections [25.0%]), skin and soft tissue infections (7 infections [19.4%]), and lower respiratory infections (6 infections [16.7%]). We found no difference in clinically-documented infections by study group. There were 45 acute care rehospitalizations, including 19 (42.2%) at intervention NHs and 26 (57.8%) at control NHs, and there were 5 deaths, including 3 (60.0%) at intervention NHs and 2 (40.0%) at control NHs. These outcomes were considered unrelated to the study. There were no adverse events related to chlorhexidine bathing, including no reported adverse skin conditions or allergic events. Additionally, the intervention did not change the number of progress notes written in the patient’s medical records nor change the number of therapy sessions (eTable 8 in Supplement 2). Participants in both groups reported visiting the dining room, therapy, and hallway preceding their study visits, with no significant differences in activities identified across study groups (eTable 9 in Supplement 2) or hand contamination after select activities (eTable 10 in Supplement 2).

## Discussion

In this cluster randomized clinical trial consisting predominantly of short-stay patients receiving postacute care at 6 NHs, we found that a multicomponent infection prevention intervention involving enhanced barrier precautions for patients with high risk, chlorhexidine bathing, hand hygiene promotion, staff education, and outcome feedback reduced overall prevalence of MDRO, with marked reductions in room environment contamination with any MDRO and VRE. Under joint analysis of patient-environment dyads, we found that this intervention significantly reduced the odds of overall MDRO prevalence in patients and their room environment.

Our study builds on previous interventions to address complex infection prevention concerns. Patient decolonization interventions can reduce bacterial load, lessen bacterial shedding, and minimize subsequent infection risk.^15,16,17,26,27^ Chlorhexidine bathing is of particular interest when designing decolonization interventions, given its low toxic effects, prolonged residual effect, and proven efficacy against nosocomial pathogens, albeit amidst emerging reports of chlorhexidine resistance.^26,27,28,29,30,31^ Chlorhexidine has been well studied in hospitals, and interventions have reduced colonization and infection with MRSA and VRE.^15,26,27,30,32,33,34,35^ Chlorhexidine-based interventions have also been shown to reduce HCW hand contamination,^34^ reduce health care–associated infections,^30,32,35^ and reduce shedding.^27^ The use of enhanced barrier precautions in NHs, markedly gown and glove use, has been reported to reduce MDRO transmission during high-risk activities, including the care of patients who are functionally dependent or who have indwelling devices.^22,36,37,38^ HCW and patient hand hygiene are important leverage points for the NH infection preventionist. HCW hand hygiene has an important impact on MDRO prevalence,^22,39^ infection rates,^40^ and outbreak management.^41^ Patient hand hygiene interventions are an emerging infection prevention tool.^42,43^ Interactive education and community-building interventions enhance the potency of other strategies. Leveraging these observations, we designed a multicomponent intervention because this approach is shown to be more effective than individual interventions.^44^

The evaluation of the impact of multimodal infection prevention interventions on environmental contamination outcomes is both important and currently lacking in the literature. Our use of dyadic data analysis strategies to assess the intervention’s effect on the MDRO presence in the patient-environment setting is a novel technique for studying health care epidemiology and MDRO transmission. By acknowledging that patients and their environments are dyads that readily transmit to one another, our results highlight how critical it is to study interventions that impact both patient colonization and environmental contamination. Moreover, dyadic analysis harnesses the statistical power of obtaining samples from both the patient and their environment. Under this analysis, we can conclude that this intervention influences patients and their environment overall, thus reducing MDRO transmission potential within facilities. Our findings are supported by limited prior observations in hospitals that chlorhexidine interventions are associated with reduced patient shedding and environmental contamination.^27^ To our knowledge, this is one of the first studies to show that multicomponent interventions can reduce environmental contamination in NHs.

### Strengths and Limitations

Our study has several strengths. First, this study is one of the more comprehensive multicomponent infection prevention interventions in the postacute care NH setting.^22,44,45^ As such, this intervention contributes to the emerging literature on the potential of multicomponent interventions in this high-risk, MDRO-burdened health care environment. Second, the pragmatic study design and multicomponent intervention implementation accurately represent real-world infection prevention efforts, accounting for the persistent challenges inherent to these facilities. Additionally, our pragmatic cluster randomized clinical trial had a far stronger design than existing pre-post experimental studies of MDRO and infection prevention, most notably by addressing bias associated with secular time-trends, such as reductions in MDRO transmission due to ongoing, nonintervention infection prevention efforts.^20^ Third, this study evaluated the intervention’s effect on patient room environment contamination.

Our study has several limitations. First, our results may not be generalizable to other regions and types of nursing facilities. It is possible that infection control and environmental cleaning practices varied between the sites; however, intervention sites received a standardized environmental cleaning education. Our intervention is resource-intensive and thus may require leadership and material support to implement. Second, future studies should extend our results to develop interventions that reduce new infections. Third, while analysis of individual organisms can assist in understanding intervention trends and mechanisms, the interpretation of the intervention’s effect on individual organisms was not the focus of the study and needs to be addressed in future work. Fourth, the enrollment rate in the intervention arm was slightly lower than the control arm, which could reflect reduced patient desire to participate in intervention procedures immediately after an acute care hospitalization. Fifth, while studies have shown the efficacy of infection prevention interventions in NHs, it is difficult to assess the effect of individual intervention components.^45^

## Conclusions

This cluster randomized clinical trial found that the comprehensive multicomponent intervention reduced the prevalence of MDROs in patients’ room environments. These findings demonstrate that multicomponent interventions can be effectively tailored to reduce the burden of MDROs in NHs.

## Notes

Supplement 1. Trial Protocol

Supplement 2. **eTable 1.** Infection Prevention Practices of Intervention and Control Group Nursing Homes **eTable 2.** Facility and Participant Characteristics **eFigure 1.** Conceptual Model Depicting the Interaction Between Environmental Contamination and Patient MDRO Burden **eTable 3.** Characteristics of Patients with at Least 1 Follow-Up Visit **eTable 4.** Microbial Swab-Level Survey of MDROs During a Multicomponent Intervention **eFigure 2.** Visit-Level Proportion of Patient Colonization and Environmental Contamination by Intervention Status **eTable 5.** Microbial Survey Results for Individual MDROs on Patients by Intervention Status **eTable 6.** Microbial Survey Results for Individual MDROs on High-Touch Environmental Surfaces by Intervention Status **eTable 7.** Onset of New Physician-Documented Infections **eTable 8.** Impact of the Multicomponent Intervention on Quality of Care **eTable 9.** Distribution of Patient Activities in the 30 Minutes Preceding the Study Visit by Intervention Status **eTable 10.** Patient Hand Contamination with Any MDRO by Patient Activities in the 30 Minutes Preceding the Study Visit by Intervention Status

Supplement 3. Data Sharing Statement
