# Supplementary material for: Effectiveness of a Multicomponent Intervention to Reduce Multidrug-Resistant Organisms in Nursing Homes: A Cluster Randomized Clinical Trial
Source: JAMA Netw Open. 2021 Jul 16;4(7):e2116555. doi: 10.1001/jamanetworkopen.2021.16555 (PMC8285736; doi:10.1001/jamanetworkopen.2021.16555)
Supplement: Supplement 1. — Trial Protocol [file jamanetwopen-e2116555-s001.pdf]

PROTOCOL NAME:

Pathway from Functional Disability to Antimicrobial Resistance in Nursing Home Patients

UM IRB protocol number: HUM00073813

ClinicalTrials.Gov registration number: NCT02909946

PROTOCOL VERSION NUMBER: 1.7

PROTOCOL VERSION DATE: 2/12/2021

GENERAL INFORMATION

Name and address of the study monitor and person authorized to sign the protocol and amendments:

Lona Mody, MD, MSc
Department of Internal Medicine, University of Michigan
Geriatric Research Education and Clinical Center
300 N. Ingalls Building, Rm 905
Ann Arbor, MI 48109.
Phone: (734) 764-8942
Fax : (734) 936-2116
Email : lonamody@med.umich.edu

Commonly Used Abbreviations:

MDRO	Multidrug-resistant organism
MRSA	Methicillin-resistant *Staphylococcus aureus*
VRE	Vancomycin-resistant *Enterococci*
GNB	Gram-negative bacilli
R-GNB	Resistant-Gram-negative bacilli
HCW	Health care worker
NH	Nursing home
HAIs	Healthcare-associated infections

1. Background

1.1 Rationale

Multidrug-resistant organisms (MDROs) are endemic in nursing homes (NHs) with prevalence rates surpassing those in hospitals. Functional disability (in activities of daily living, ADLs) has been shown to be a significant risk factor for the new acquisition of MDROs, and that contact-intense ADLs, such as bathing and toileting increase this risk. We hypothesize that specific ADL disability patterns in NH patients will predict acquisition risk for new MDROs. We also theorize that disability and risk of acquisition will be proportional to contact time and contact intensity with HCWs who provide care to these patients. Greater time and intensity of contact increases the likelihood that patients will acquire MDROs through contact with the contaminated hands of HCWs and with their environment. Additionally, we hypothesize that a patient hand hygiene program may help to reduce the transmission of MDROs to environmental surfaces, HCW, and other patients. The aim of the study is to design and evaluate the effectiveness of a multi-component intervention to reduce the prevalence of MDROs and new acquisition of MDROs in all patients, including functionally disabled NH patients at highest risk as defined by a previously described risk stratification model.

1.2 Population

The study will be conducted at six NHs in southeast Michigan. Two study populations will be studied: nursing home patients and the HCWs providing formal direct care to participating patients.

1.3 Risk/Benefits

The risks related to this study are minimal. There are two types of participants: 1) NH patients and 2) HCWs.

Potential risks to all patient participants include those associated with non-invasive cultures swabs to the nares, oral, hands, groin, perirectal area, wounds and device site (if present), and the loss of confidentiality of individual health information collected as part of the study, however, these risks will be rare.

We will minimize these risks as follows. Patient participants will be educated on how the cultures are performed at the time of enrollment to reduce any psychological discomfort. Study coordinators will have competencies in doing these procedures and will use gentle pressure to reduce possible irritation or discomfort during the culture procedures. Access to study records will be restricted to study staff and investigators. Every effort will be made to keep the study records as confidential as possible through the use of assignments of unique participant identifiers, password-protected databases and storage of paper records in locked filing cabinets in rooms that are locked when not in use (see **11. Data Collection, Handling, and Storage**).

Potential risks for patients participating in the hand hygiene survey could include feeling uncomfortable answering certain study questions out of concern that their answers could affect the care they receive while at the NH if a loss of confidentiality were to occur and their responses known. To minimize this risk, the patient will not have to answer any or all questions that they are not comfortable answering, and we will not collect patient names or identifiers which could link a survey to the patient.

The intervention aim also includes enhanced barrier precautions for high-risk patients and the use of antimicrobial bathing cloths for all patients (daily for high-risk, twice weekly for all other) enrolled at the intervention sites. We will minimize these risks as follows. We will not restrict any of their activities if they are colonized with any MDROs since we expect patients to

stay in NHs for prolonged periods of time. We will instead emphasize enhanced barrier precautions to all ancillary staff providing bathing, toileting, dressing, or grooming care to the high-risk patients.

Chlorhexidine has been widely used in healthcare for decades for skin disinfection, handwashing, oral care, irrigation of surgical wounds, and many others. Daily bathing with chlorhexidine is commonly used within the acute care hospital setting (including our own University of Michigan Health System) to disinfect the skin and decrease the transmission of nosocomial pathogens including MDROs like MRSA and VRE, and reduce infections caused by these pathogens such as central line-associated bloodstream infections, CAUTIs, surgical site infections and skin and soft-tissue infections. Prior research provides strong support for the concept of source control as a strategy to reduce the dissemination of healthcare-associated pathogens (i.e., reducing the burden of pathogens on the skin as a means to reduce dissemination to the environment or hands). Given its broad spectrum of activity, chlorhexidine bathing as a daily skin cleanser represents a horizontal infection prevention approach that can potentially reduce the dissemination of multiple pathogens, including MDROs such as MRSA, VRE, and R-GNB. In this study, the 2% chlorhexidine gluconate cloths for daily bathing will be used as one component of a multicomponent infection prevention program to reduce the prevalence and new acquisition of MDROs on patient's skin, healthcare worker hands, and the environment, and to reduce the development of new healthcare-associated infections (HAIs) in patients. One of the most common MDROs in nursing home patients is MRSA, which is a frequent cause of skin and soft tissue infections, surgical site infections, and other HAIs. Preliminary data shows that 76% of nursing home patients in our study are considered to be high-risk for MDRO colonization and infection. 2% chlorhexidine gluconate cloths have been used as a daily bathing cloth in several studies and shown to be well tolerated. Patients may experience redness, itching, dryness of the skin, irritation of the mucous membranes or allergic reaction to the chlorhexidine bathing cloths, although it is infrequent (~1%). Despite the overall low rate of expected adverse events, several restrictions will be placed for the use of the 2% Chlorhexidine Cloth. The 2% Chlorhexidine cloth will not be used in the following situations:

1. On patients with known allergies to chlorhexidine gluconate or any other ingredients in the product.
2. The product should be kept out of the eyes, ears, and mouth and open skin areas
3. For lumbar punctures or in contact with the meninges.
4. Patients with a severe skin disease or burns.

HCW will assist patients to use the chlorhexidine cloths. Serious or permanent injury could occur if chlorhexidine is permitted to enter and remain in areas such as eyes, ears, mouth, open skin, meninges, or burns. If this should occur, the area should be rinsed with cold water right away, and the patient's healthcare providers and the study coordinator/principal investigator will be notified. Patients will also be monitored for irritation or reactions during their follow-up. If such irritation or reactions occur, they will be reviewed by the study coordinators and principal investigator. Discontinuation of the cloths may be implemented in consultation with the Principal Investigator/Study Coordinators.

To ensure that the intervention program does not adversely affect the quality of enrolled patient's care, we will monitor patient's responses to questions about their perceived quality of care while at the nursing home to assess for any systematic differences between intervention and control facilities.

Potential risks for HCWs include those associated with the non-invasive culturing of hands and clothing, use of enhanced barrier precautions, educational interventions and loss of confidentiality. HCWs will wear disposable gowns and non-latex gloves while providing care for

the enrolled patients at intervention sites. Wearing gowns and gloves are part of Standard (infection control) Precautions for certain care activities and offer no additional risk to the HCWs. HCWs may experience some drying of their hands with repeated hand hygiene. We will ask HCWs to report any instances of dryness to the study team and will suggest adequate moisturizing lotions. Potential risks to the HCWs as they attend in-services and answer knowledge tests will be minimal since almost all of the questions will focus on infection control practices. Some questions pertaining to knowledge regarding hand hygiene, indwelling devices, or environmental cleaning may be perceived to be intrusive. Random observations of HCWs as they perform device care may be also be perceived as intrusive. We will structure our questions and observations to minimize such intrusiveness. We will also ensure HCWs of confidentiality. While we will collect demographic data and identifiers from each HCW; only aggregate data on compliance and adherence will be reported to the facility leadership.

There is no direct, individual benefit that can be guaranteed as a result of participation in this research study. There is a societal benefit, as this study will provide information about the transmission of bacteria that could ultimately reduce the risk of bacterial infection for other NH patients. All patients, HCWs and family/visitors will be given the opportunity to have their questions answered by the study coordinators or investigators as needed. Given the minimal risks to the participants, the benefits outweigh the risks.

1.4 Study Conduct

This study will be conducted in compliance with the current protocol approved by the Institutional Review Board and according to Good Clinical Practice standards. All study documents will be located on a restricted access shared folder on the university server. The current protocol will also be posted there. E-mail alerts to all study personnel will be generated with each change in the version of the protocol. These will include a summary of the modifications. All approval letters from local Institutional Review Boards will be posted on the folder. Current consent forms will also be posted. No deviation from the protocol will be knowingly implemented without the prior review and approval of the IRB except where it may be necessary to eliminate an immediate hazard to a research subject. In such a case, the deviation will be reported to the IRB as soon as possible.

2. Study Aims

We will use a previously-defined risk-stratification model to inform our study's aim, which is to design and evaluate the effectiveness of a multi-component intervention to reduce the prevalence of MDROs and new acquisition of MDROs in all patients, including functionally-disabled NH patients at highest risk as defined by the risk stratification model.

3. Study Design

3.1 Primary Study Endpoints

The primary study endpoint for this study is the prevalence of MDROs, and the new acquisition of an MDRO by a patient participant. Additionally, we will assess incident healthcare-associated infections; environmental transmission; healthcare worker knowledge pertaining to infection control topics; patient knowledge, experiences and preferences for hand hygiene; and enhanced standard precaution compliance for hand hygiene, gown and glove use.

3.2 Study Design/Type

We will develop and test an intervention for all patients, with a focus on functionally disabled NH patients at the highest risk of acquiring an MDRO. Three facilities will be randomized to the

intervention and three will serve as control sites. Data gathered from our previously defined risk-stratification model will be crucial in informing the population to target for this intervention, the role of specific types of ADL activities in increasing transmission and persistence of MDROs, as well as an intervention to lower MDRO acquisition, transmission, and colonization in NHs. The intervention will incorporate caregiver, patient-level, environmental, and facility-level strategies and includes:

1. Standard precautions and hand hygiene for HCW for care of all patients. Enhanced barrier precautions including hand hygiene, glove use, and gown use for HCW when providing ADL assistance to high-risk patients (HCW intervention).
2. Hand hygiene survey for patients, and hand hygiene education to patients and families (patient-level intervention).
3. Standardized bathing practices including using chlorhexidine-based cloths to reduce patient MDRO colonization. High-risk patients will receive daily bathing with chlorhexidine-based cloths, all other enrolled patients will receive twice-weekly bathing with chlorhexidine-based cloths (patient-level intervention).
4. Standardized environmental protocol and education to reduce contamination on inanimate surfaces (environmental intervention).
5. Feedback monthly of facility-level microbial data including MDRO prevalence and new MDRO acquisition rates, and process measures such as hand hygiene, gown and glove use to infection control practitioners, HCWs, and facility leadership (facility intervention).

Enrolled patients will be assigned to the high-risk category for acquiring an MDRO based on a previously described risk-stratification model:

- Functional disability: patients needing moderate to total assistance with dressing, bathing, toileting, or grooming.
- Presence of indwelling devices: urinary catheter and/or feeding tube.
- Presence of a wound: open chronic wound requiring regular dressing changes by nursing staff or requiring a wound vacuum.

3.3 Duration

Enrolled patients will receive study visits at baseline, at 7 days, 14 days, 21 days, 30 days, and monthly thereafter for up 6 months. Each patient will be followed from the enrollment through a minimum of 7 days to a maximum of six months, death, or discharge from the facility.

4. Selection and Withdrawal of Participants

4.1 Inclusion Criteria for Patients

Multicomponent Infection Control Intervention:

- Age ≥18 years
- Reside in a participating NH facility
- Written informed consent from participant, or written informed consent from LAR with assent from participant, or written informed consent from LAR with waiver of assent for those not able to assent

4.2 Exclusion Criteria for Patients

- Patients receiving end-of-life care
- Non-English language speaking

4.3 Inclusion Criteria for HCWs

- Health care worker at participating NH site
- Has direct interaction with participating patients at study site
- Informed consent

4.4 Exclusion Criteria for HCWs

- None

4.5 Subject Withdrawal

Study patients or HCWs will be withdrawn from the study if found to have been initially ineligible or if consent is withdrawn. No follow-up with withdrawn participants is required.

4.6 Medication

All medications are permitted during the study.

5. Facility Recruitment

Patients and healthcare workers will be recruited at participating NH facilities. Prior to enrollment, the PI will meet with the facility administrators, including the medical and nursing directors at each NH to review the study aims and protocols.

6. Informed Consent for Patients

6.1 Patient Recruitment

We are requesting a <u>partial consent waiver for recruitment</u> to allow the study coordinator to review the medical record of potential participants to determine initial eligibility, medical decision-making capacity, and contact information for the next of kin or legally authorized representative. Accessing this information from the patient's medical record reduces the burden on the patient with regard to time and research procedures, increases the feasibility of the study, and does not confer any additional risk.

6.2 Informed Consent Process for Patients

1. Informed Consent – Patient eligible and able to give consent
2. Informed Consent by LAR +Assent – Patient eligible, not able to give consent but is alert and able to communicate, patient LAR gives signed consent or telephonic consent, patient able to assent
3. Informed Consent by LAR + Waiver of Assent – Patient eligible, not able to give consent and is not alert or able to communicate, patient LAR gives signed consent or telephonic consent, request waiver of assent from patient

The study coordinator will tell the patient about the study. If the patient expresses interest in the study and is willing to participate, the study coordinator will verbally review the information on the <u>IRB approved Consent Form</u> with the patient in a private area. The patient will be asked if he/she wishes for the study coordinator to read the consent form verbatim or to summarize it as the patient follows along.

Because cognitive impairment is common in the nursing home population, the patient will be evaluated for his/her ability to give informed consent. The patient must be alert and able to communicate in order to give informed consent. If the potential participant is considered competent to consent, he/she will sign his/her name on the study's copy of the Informed

Consent Form. The patient will receive a copy of the signed Consent Forms, which will be placed in the patient's NH medical record.

If the potential participant is considered incompetent to consent and his/her legally authorized representative (LAR) [this may be a durable power of attorney for healthcare (DPOA) or guardian for the patient] as listed in the medical record will decide whether it is in his/her best interest to participate in the study. If the LAR is available at the LTCF, then the study coordinator will review the information from the Consent Form with them and they will have the opportunity to have his/her questions answered. The LAR will sign his/her name on the Consent Form. A copy of the signed forms will be placed in the patient's NH medical record.

If the LAR is not available at the LTCF, then they will be contacted by telephone to discuss the study using the Telephonic Consent from LAR Script and will be given the same opportunity to have his/her questions answered. We are requesting a Waiver of Documentation of Consent for LARs that are not available to sign the Consent Form. The study coordinator will document the name of the person granting consent, relationship to the patient and the date consent was obtained on the study's copy of the Consent Form. A copy of the signed Consent Form will be placed in the patient's NH medical chart.

Participant assent to study procedures will be obtained prior to any procedures for those patients that are deemed unable to consent for themselves and consent of a LAR is obtained. Prior to study procedures, the study coordinator will briefly explain the study purpose (degree of explanation will depend on the patient's level of cognition) and study procedures (Assent form). The participant will assent by verbal agreement or positive gesture. If the patient is able, he/she will sign the Assent Form. If the patient does not assent to study procedures, no further procedures will be performed. For patients not able to assent, but consent has been obtained from the LAR, a Waiver of Assent is requested.

6.3 Informed Consent Process for Patient Hand Hygiene Survey Only

The study coordinator will tell the patient about the survey study. If the patient expresses interest in the study, the study coordinator will provide the patient with a copy of the Consent Form and a survey. The patient will be asked if he/she wishes for the study coordinator to read the consent form verbatim or to summarize it as the patient follows along.

Because cognitive impairment is common in the nursing home population, the patient will be evaluated for his/her ability to give informed consent. The patient must be alert and able to communicate in order to give informed consent. Patients unable to provide informed consent will not be approached for the survey. We are requesting a waiver of written documentation of informed consent to assure that the patient would not feel that if the researchers have the names of the participants it could affect their care at the nursing home if a loss of confidentiality were to occur and their individual responses were known. The consent form would be the only study document that would link the patient to the research survey. We will not be requesting the names of participants or reporting to the facility who chooses or refuses to participate.

7. Informed Consent for Health Care Workers (HCWs)

7.1 Recruitment Process

HCWs will be recruited at the participating NH facilities. Prior to study commencement at a NH, a meeting will be scheduled with facility/unit administrators including the Medical Director and Director of Nursing at each prospective NH. Upon confirming their agreement to participate, we will meet with unit nurse managers and medical providers. We will also present at any staff meetings to explain study procedures, answer questions, and address concerns. In order to

assure that HCWs are not coerced into participating, we will not report participation to supervisors. There will be no penalties or loss of benefits for not participating.

7.2 Informed Consent Process for Health Care Workers

The HCW will be told about the study by the study coordinator following the Consent Form for healthcare workers. If the HCW is willing to participate, the study staff will provide a copy of the informed consent. The HCW will not be required to sign the informed consent document, as this is the only study document that will link the HCW to the research study. We are requesting a waiver of written documentation of informed consent to assure that the HCW would not be coerced into participating by feeling as though their refusal to participate or by feeling that if the researchers have the names of the participants it could affect their job status with their employer if a loss of confidentiality were to occur. Therefore, we will not be requesting the names of participants or reporting to their supervisors or the facility who chooses or refuses to participate.

8. Study Procedures

8.1 Patient Cultures

At enrollment and each follow-up visit, trained study coordinators will collect samples from patients of the anterior nares, oral, groin, perirectal skin, wounds, and device sites (if present) for bacteria using a culture swab. These samples will be plated onto Bile Esculin Agar containing 6 g/mL vancomycin, Mannitol Salt Agar, and MacConkey Agar, and assessed for the presence of MRSA, VRE, and R-GNB utilizing standard microbiology testing methods. Patient hands will also be cultured using the procedure outlined in 8.3 below.

8.2 Environmental Cultures

To assess the amount of environmental contamination of the patient’s immediate environment, cultures will be collected at each patient study visit. Pre-moistened culture swabs will be applied to 5x20-cm areas of a patient's bed rail, bedside table, undersurface near the edges (most likely to be grabbed but not cleaned), door knobs, toilet seats, charts, patient equipment such as intravenous pump, high-touch curtain areas, call button, and telephone. Approximately ten environmental swabs from each patient’s room will be collected at each patient study visit. Approximately ten environmental swabs from common use areas such as dining rooms, living rooms, rehab gym, and common equipment will be obtained from each facility monthly. These samples will be swabbed onto Bile Esculin Agar containing 6 g/mL vancomycin, Mannitol Salt Agar, and MacConkey Agar and phenotypically unique colonies will be identified by standard methods.

8.3 Multi-component Infection Control Intervention

8.3.1 Randomization Procedure:

We will randomize NH facilities instead of participants using cluster randomization as randomizing participants can lead to contamination by HCW experiences learned while caring for the intervention group. Similarly, randomizing individual HCWs can also introduce bias due to contamination. Therefore, NHs will serve as the unit of randomization. We will enroll six facilities; three will be randomized to the intervention group and three to routine care (control group). The randomization procedure will be conducted by the study statistician and concealed to study personnel responsible for the fieldwork.

8.3.2 Component 1: Caregiver Intervention

8.3.2.1 Standard Precautions and Hand Hygiene

Standard precautions will include hand hygiene by HCW before and after providing any care for all patients. Gloves and gowns will be used by HCW during care activities that may cause hand contamination, or splashes onto HCW clothing, respectively.

Formal (Nurses, nurses' aides, rehabilitation personnel, recreation therapists, dietary services, environmental) and informal caregivers (family and visitors) will be in-serviced on indications for hand hygiene, glove and gown use when providing care for all patients. Tools such as personalized posters, demonstration of hand hygiene techniques, and simplified tailored infection prevention education will be provided. Structured observations will be conducted to monitor compliance.

We will discuss our aggregated results with HCWs during their in-services, with infection control practitioners and their administrators at monthly meetings, as well as other clinical providers, such as physicians, nurse-practitioners, and physical and occupational therapists during their in-services.

8.3.2.2 Enhanced Barrier Precautions

Enhanced barrier precautions will include hand hygiene, glove use, and gown use for HCW when providing ADL assistance, care for indwelling devices, or wound care to high-risk patients.

All high-risk patient participants will be placed on enhanced barrier precautions including:

1. 'High-risk' signage in charts and electronic medical records;
2. Appropriate hand hygiene before and after providing any care;
3. Gloves to be worn when providing any assistance with bathing, dressing, grooming, toileting, device care, or wound care, in addition to times indicated per Standard Precautions. Appropriate hand hygiene to be performed before and after wearing gloves; and
4. Protective gowns to be worn as part of barrier precautions when providing any assistance with bathing, dressing, grooming, toileting, device care, or wound care, in addition to times indicated per Standard Precautions.

Formal (Nurses, nurses' aides, rehabilitation personnel, recreation therapists, dietary services, environmental) and informal caregivers (family and visitors) will be in-serviced on indications for hand hygiene, glove and gown use when providing care for their high-risk patients. Tools such as personalized posters, demonstration of hand hygiene techniques, and simplified tailored infection prevention education will be provided. Structured observations will be conducted to monitor compliance.

We will discuss our aggregated results with HCWs during their in-services, infection control practitioners, and their administrators at monthly meetings, as well as other clinical providers, such as physicians, nurse-practitioners, and physical and occupational therapists during their in-services. We will not isolate participating patients to their rooms; they will be allowed to socialize and get rehabilitation. This approach is practical and safer for our older NH patients, allows them to socialize and get their rehabilitation, while at the same time intervenes to stop acquisition and spread of MDROs.

8.3.3 Component 2: Patient Intervention

8.3.3.1 Standard Precautions and Hand Hygiene

We will conduct a short survey of patients on their knowledge, experiences, and preferences in cleaning their hands while in the NH. We will conduct the survey at the start of the intervention period. The survey information will be used to design a patient hand hygiene promotion program in intervention facilities, as discussed below. We may conduct the survey again at 12 months, and study end to determine if the patient hand hygiene program changed patient's knowledge, experiences, or preferences regarding hand hygiene.

8.3.3.2 Hand Hygiene Education

We will conduct an active hand hygiene campaign for enrolled patients which may include a) education materials targeted to patients; b) reminders for the NH staff to assist patients with hand hygiene at key times; and c) providing soap/water or hand sanitizing wipes to patients to use for hand hygiene. Education materials will be targeted to patients using CDC-approved educational posters promoting patient use of hand sanitizing wipes or antimicrobial soap and water for hand hygiene for all approved indications such as before and after touching wounds or devices, before meals, after using the bathroom, as well as before leaving their rooms, using the common areas, and participating in group activities. Structured observations will be conducted periodically to monitor compliance.

8.3.3.3 Standardized Patient Bathing

Chlorhexidine-based cloths have been shown to reduce skin colonization with MDROs. Enrolled patients will receive a bath using 2% chlorhexidine gluconate cloths. High-risk patients will receive daily bathing using chlorhexidine bathing cloths, while all other enrolled patients will receive a bath at least twice weekly using chlorhexidine bathing cloths at their usually assigned shower times. The bathing frequencies were chosen to coincide with routine times when an enrolled patient would be expected to receive significant NH caregiver assistance. NH staff will be trained using a standardized protocol to perform daily or twice-weekly bathing using the chlorhexidine cloths. Briefly, the CHG cloths are packaged with 6 cloths per pack. One cloth each is used on a different body site using a firm massage: 1) neck shoulders and chest, 2) both arms and hands, 3) abdomen, then groin, then perineum, 4) right leg and foot, 5) left leg and foot, 6) back and buttocks. The cloths will not be used on the face, open wounds, burns, meninges, mucous membranes, or on patients with known allergies to CHG. For patients with incontinence, the urine and stool will be removed using the facilities usual procedure, followed by cleaning with a CHG cloth. For patients with catheters, after cleaning the skin the 6 inches of tubing nearest the patient can be cleaned. For patients with open wounds covered with a dressing, the cloth can be used to clean the skin up to the dressing. Compliance monitoring will be completed by 1) periodic observations of patient bathing with staff assistance to ensure adherence to the bathing protocol, 2) weekly site visits to monitor the amount of bathing cloths used and ensure adequate supply exists. Many skincare moisturizers that contain anionic emulsifiers may adversely affect the residual antibacterial effect of chlorhexidine and should be avoided during routine care during periods in which chlorhexidine is in use. We will work with each NH to ensure appropriate skincare products are available.

8.3.3.4 Clinical Pathways for Antibiotic Utilization

We aim to enhance appropriate antibiotic usage utilizing clinical pathways to reduce antibiotic use in NHs. HCW will be educated on standardized surveillance definitions and minimum criteria for initiation of antibiotics for common infections in NHs at the intervention sites, which may be implemented using pocket-card handouts with infection definitions, small group

interactive sessions for caregivers, videos, outreach visits, and one-on-one visits with physicians providing care at these three sites.

8.3.4 Component 3: Environmental Intervention

The main goal of this component is to reduce contamination on inanimate objects and surfaces in patient rooms MDRO. High-touch target areas will be cultured and evaluated for baseline cleaning effectiveness. Additionally, visual inspection and removal of fluorescent markers may also be used to assess the thoroughness of cleaning. These results will be shared with facility leadership and environmental services. A standardized educational program targeted to environmental services personnel will be designed for the intervention sites. The content will include the importance of disinfection and cleaning for patient safety, environmental personnel, and HCWs; review of different products to use; and standardized cleaning protocol for the environment, including cleaning and disinfection of high-touch surfaces in patient rooms and common areas at least daily. Environmental services personnel at the intervention facilities will be asked to attend five 30-minute sessions over 24 months. Each session will be offered at least twice during the day and evening shifts to include night shifts. A knowledge survey may be administered pre- and post-session to determine baseline knowledge levels and effectiveness of the education program. One-on-one education and feedback on performance may also be given to environmental services personnel throughout the intervention period at intervention facilities. Posters and checklist materials may be used to increase compliance with cleaning procedures.

8.3.5 Component 4: Facility Feedback

We will generate monthly reports to give feedback on the a) facility-level microbial data including: MDRO prevalence and new MDRO acquisition rates; and b) process measures including: hand hygiene, gown and glove use, and chlorhexidine bathing compliance to infection control practitioners, HCWs, and facility leadership. Graphs, charts, and tables easily understood by HCWs will be used to present the data. The ICPs will be encouraged to share the data with their nurses, nurses' aides, therapists, environmental services, and visitors. These reports will provide aggregate results and will not identify individual patients, HCWs or family/visitors. In addition, all NH staff will be invited to attend five 30-minute sessions over 24 months. Feedback of study data to-date will be provided at each session, as well as education regarding study concepts (such as hand hygiene, PPE use, device care, MDROs, etc.), and review of study protocols. Each session will be offered at least twice during the day and evening shifts to include night shifts. A knowledge survey on study concepts and protocols may be administered pre- and post-session to determine baseline knowledge levels and effectiveness of the education program. One-on-one education and feedback on performance may also be given to NH staff throughout the intervention period at intervention facilities.

Process outcomes for hand hygiene, glove use and gown use, and chlorhexidine bathing compliance will be measured by direct observation of HCWs providing ADL assistance, device or wound care, and bathing.

The educational outcomes of our intervention will be assessed using two separate tools. First, for each in-service, a survey consisting of 10-15 objective questions will be conducted. The test will be piloted first to examine validity and internal reliability (using Cronbach's coefficient alpha). Differences between the intervention and control caregivers will be calculated. Second, a course evaluation may be conducted to assess the relevancy and usability of the material, the teaching effectiveness of the trainers, the quality of individual sessions, course handouts, as well as the DVD. Open-ended questions will be posed to the participants to express any other comments or concerns. The infection control practitioner at

intervention sites will also be introduced to other resources from various national infection prevention societies.

Because of the nature of the intervention, it cannot be blinded to the patients, caregivers or investigators. However, our microbiology laboratory personnel assessing the outcomes will be blinded to the assignment of the facility treatment arms.

8.4 Study Variables

Exposure data on demographic characteristics, co-morbidities, length of stay, cognitive status, antibiotic usage (type and duration), hospitalization along with diagnoses at transfer, presence of wounds including pressure ulcers, falls, and antipsychotic use will be obtained [Table 1]. Facility-level data such as staffing patterns, bathing practices, cohorting based on risk factors, presence of shared and private rooms, frequency, and room-cleaning time will also be collected. Outcome data on the presence of MDROs, infection control knowledge, and adherence to standard and enhanced standard precautions, hand hygiene, and chlorhexidine bathing will be collected as described above.

Table 1. Study variables

Variable	Source of Information*	Times of Assessment†
**Patient Descriptors**		
Demographics	I, C	B
Duration of stay at the facility	I, C	B
Visit number	SD	B, d7, 14, 21, 30, q 30d
**Primary Exposure Measurements**		
Functional Status using Physical Self-Maintenance scale	I, C	B
Wounds, hospitalization, antibiotic use, device use	I,C	B
**Exposure Measures: Room Environment Cultures**	RM	B, d7, 14, 21, 30, q 30d
**Primary Outcome: MDRO Prevalence**		
MRSA and/or VRE and/or R-GNB	RM	B, d7, 14, 21, 30, q 30d
**Secondary Outcome: New MDRO Acquisition**		
MRSA and/or VRE and/or R-GNB	RM	B, d7, 14, 21, 30, q 30d
**Exploratory Outcome: Healthcare Associated Infections**		
Clinically-defined infections (e.g., UTI, Pneumonia, skin & soft tissue infections)		B, d7, 14, 21, 30, q 30
**Other Patient Risk Factors and Predictors**		
Comorbidity, Charlson's index	I, C	B

* Source of information: I, Interview (patient and/or proxy); C, medical chart review including all nurses, aides, physicians, rehabilitation notes, clinical laboratory data, pharmacy data, hospital transfer data); SD, study tracking data; RM, research microbiology data.

† Times of assessment: B, baseline; q 30d, every 30 days until 6 months or discharge.

9. Adverse Events

9.1 Adverse Events and their Grading

We will collect data on serious adverse events (SAE) that occur while the study participants are enrolled. Elderly nursing home patients are at a particularly high-risk for adverse events, including falls, infections, hospitalizations, and death. We will monitor for all SAE that are possibly related, probably related or definitely related to our research protocol. A serious adverse event is any adverse experience that results in any of the following outcomes: Death, a life-threatening adverse drug experience, inpatient hospitalization or prolongation of existing hospitalization, a persistent or significant disability/incapacity, or a congenital anomaly/birth defect. Important medical events that may not result in death, be life-threatening, or require

hospitalization may be considered a serious adverse drug experience when, based upon appropriate medical judgment, they may jeopardize the patient or participant and may require medical or surgical intervention to prevent one of the outcomes listed in this definition.

The severity of the adverse events will be defined as follows:

- 0 – No adverse event or within normal limits
- 1 – Mild – awareness of sign, symptom or event but easily tolerated
- 2 – Moderate – discomfort enough to cause interference in usual activity and may warrant intervention
- 3 – Severe – incapacitating in ability to do usual activities or significantly affects clinical status, and warrants intervention
- 4 – Life threatening or disabling AE
- 5 – Fata AE

9.2 Attribution of Adverse Events

The Principal Investigator will determine attribution of serious adverse events. The following scale will assess the relationship of AEs to the study procedures:

- Not related: no temporal association, or the cause of the event has been identified, or the study procedures cannot be implicated
- Possibly related: temporal association, but other etiologies are likely to be the cause; however, involvement of the study procedures cannot be excluded
- Probably related: temporal association, other etiologies are possible but unlikely
- Definitely Related: established temporal or other association for event not reasonably explained by the patient's known clinical state or any other factor

The Principal Investigator will also determine the expectedness of the serious adverse event as follows:

- Expected: The event is expected if it has been addressed in one or more of following: Protocol, Investigator Brochure, Package Insert or equivalent, published literature, IRB application, grant application, Data Safety Monitoring Board/Data Safety Committee reports, other documentation, informed consent document (ICD) or characteristics of the study population, the natural progression of any underlying disease, disorder, or condition of the subject(s) experiencing the adverse event.
- Unexpected: The event has not been addressed in one or more of the above examples.

9.3 Adverse Event Reporting

SAEs that are possibly, probably or definitely related to the research and are unexpected in nature will be reported to the IRB as soon as possible and no later than specified by IRB policy for standard adverse event reporting. The principal investigator will evaluate the adverse event and determine whether the adverse event affects the Risk/Benefit ratio of the study and whether modifications to the protocol or consent form are required. If an adverse event leads to a modification of the protocol or a consent form, access to the consent forms will be locked and an e-mail alert sent to all study personnel.

The principal investigator will conduct a review of all serious adverse events annually. The principal investigator will evaluate the frequency and severity of the serious adverse events and determine if modifications to the protocol or consent form are required. A summary of the serious adverse events will be reported to the IRB annually, when renewal is sought.

10. Statistical Plan

10.1 Data Analysis: Multicomponent Intervention

For this study, we will evaluate the association between receiving the multicomponent intervention and MDRO prevalence. To evaluate the impact of this intervention on MDRO prevalence, we will use univariable and General mixed effect models with multivariable logistic regression to predict the presence of each MDRO individually (MRSA, VRE, and R-GNBs). We will additionally predict the presence of any of the three MDROs. This analysis will be done separately for the patient and environment specimens. Analysis will be conducted on specimen-level and visit-level data.

Patient and environment visit-level data will be combined as a composite outcome. We will use generalized mixed effect modeling with logistic regression. To assist in the modeling of dyadic outcomes (e.g., MDRO prevalence in both patient and environment samples), we will adjust for multilevel data by considering two random effects, the dyad and the patient/environment within the dyad. This will be performed to account for the potential that an intervention can influence both a patient and his/her partner (e.g., patient room environment samples).

To evaluate the impact of this intervention on MDRO acquisition, we will test subsamples of patients with more than one sampling visit. These subsamples will only include patients who are free of the MDRO of interest (e.g., MRSA, VRE, R-GNB) on admission. We will use univariable and multivariable Cox regression modeling to predict the acquisition for new acquisition of each MDRO and the combined MDRO outcome. Given our interest in environmental contamination, we will additionally include patient room environment contamination data into the multivariable Cox regression model to identify whether the identify effect holds after accounting for the influence of the patient room on MDRO transmission. Acquisition rates will be calculated and defined as new acquisition events per 1,000 patient-days.

For our exploratory analyses, we intend to collect data on the onset of physician-defined onset of new healthcare-associated infections. Although this study was not powered to test for the intervention effect on infection data, we intend to report the longitudinal patterns on patients and compare infection rates.

10.2 Sample Size/Power Estimates

Based on a previously described risk-stratification model, we expect to identify patients at the highest risk of new acquisition. We expect this pool to be 955 (44% of all new admits) from six facilities over two years. We assume that 50% of patients or their DPOA will give informed consent (=478). Our previously conducted risk stratification analyses call for at least a 90-day follow-up. We expect 50% of these patients to stay more than 90 days, giving estimated high-risk study population to be 239 patients in all six NHs or 119/each cohort enrolled over two years and followed up to six months. Based on a 42% reduction in MRSA transmission in acute care, we expect to have a conservative 30% reduction in new MDRO acquisition as a result of our intervention. The expected proportion of new MDRO acquisition (π_0) in the high-risk *control* group is 0.6. The proportion of new MDRO acquisition in the high-risk *intervention* group with 30% reduction (π_1) is 0.42. The desired power of the study is 80% (z_2 = 0.84); the desired significance level is 0.05 (z_1 = 1.96).

Since our study is a prospective cohort study of patients in clusters at each NH, we need to adjust for intracluster correlation. As per Donner and Klar 2004 and Hayes and Bennett 1999 suppose that we have to randomize *c* clusters (i.e., NHs) to each group *i* where *i* = 1 for the

intervention group and i = 0 for the control group. We are estimating a conservative average of 119/cohort enrolled over two years. K denotes the coefficient of variance of true proportions between clusters within each group. We will take the conservative assumed value to be at 0.07 (varies between 0.03-0.1 in NH studies) [Table 2]. A cluster study from Loeb et al. 2006 using clinical pathway to reduce NH pneumonias used a k of 0.04.

Table 2. Sample Size Calculations with Values of Intracluster Coefficients and Reduction in MDROs

K	% Reduction	NH/cluster
0.06	25	3.13
0.06	30	2.45
0.06	35	2.04
0.07	25	3.38
**0.07**	**30**	**2.62**
0.07	35	2.16
0.07	40	1.86
0.08	25	3.68
0.08	30	2.81
0.08	35	2.29

The required sample size c is thus given by:

$$c = 1 + \frac{(z_1 + z_2)^2 \left[\pi_0 (1-\pi_0)/n + \pi_1 (1-\pi_1)/n + k^2 (\pi_0^2 + \pi_1^2)\right]}{(\pi_0 - \pi_1)^2}$$

$$c = 1 + \frac{(1.96 + 0.84)^2 \left[0.24/119 + 0.2436/119 + 0.07^2 (0.6^2 + 0.42^2)\right]}{(0.6 - 0.42)^2}$$

=1 + 1.619 or 2.62 NHs/group or 3 NHs/group

Thus, we will enroll three facilities per group.

11. Data Collection, Handling and Storage

The Principal Investigator is responsible to ensure the accuracy, completeness, legibility, and timeliness of the data reported. Data collection is the responsibility of the study staff.

Paper records or case report forms will be filled out at the participating community-based NH. Copies of the paper records or case report forms will serve as source documents and maintained for recording data for each subject enrolled in the study. All source documents will be completed in a legible manner to ensure accurate interpretation of data. Black ink is required to ensure clarity of reproduced copies. When making changes or corrections, the original entry will be crossed out with a single line, and the change initialed and dated. Erasing, overwriting, or use of correction fluid or tape will not be done.

All source documents and laboratory reports will be reviewed by the clinical team and data entry staff, who will ensure that they are accurate and complete. AEs must be graded, assessed for severity and causality, and reviewed by the PI or designee.

Confidentiality will be maintained to the fullest extent permitted by law. All clinical data will be collected on case report forms that will be scanned into a password protected relational database which will be kept on a research server that has a level and scope of security that

equals or exceeds that established by the HIPAA Security Rules. Paper records will be stored in locked filing cabinets in a protected space. Data will be maintained on a secure electronic central database on a research server. Data will be backed up according to the network backup schedule.

A unique identifier or code will be assigned to each patient participant. Bacterial isolates and culture specimens and datasets used for analysis will be labeled with this code. Only the study personnel who directly interact with the subject or manage the subject's clinical protocol data will have access to participant identifying information. Culture data will be entered directly into the relational database; however, microbiology laboratory personnel will not have access to participant identifiers in the database. Study personnel who leave the research team will have their access to study data removed.

As soon as permitted and when all data analyses are complete and have been published, source documents containing identifiable information will be shredded and identifying information will be removed from the database. During the entire study, all data will be managed centrally at the primary site. There will be a single data table that maintains the link between the unique code and patient identifiers. This table containing the link between code and identifying information will be deleted on the server and in any backups when it is time to deidentify the database. If any paper copies of the link have been made, they will be shredded.

12. Study Management Plan

12.1 Study Coordination

The University of Michigan will be the coordinating site for this study under the direction of the PI, Dr. Mody. It will implement the protocol in community-based NH in Michigan. We will coordinate study operations, maintain the current version of study documents, and be the central repositories for data entry into a centralized database, and perform all of the microbiology testing. We will provide updates to all study NH sites during the entire study period. These updates will cover study progress, summarize changes in the protocol, identify operational changes, and disseminate interim results.

12.2 Performance Monitoring:

The performance of each NH site will be monitored via the centralized study database. The monitoring period will be specific to each study site, beginning soon after enrollment begins at the site, and continuing through site data collection closeout. Monitoring will focus on timing, frequency and quality of study recruitment, follow-up, and data collection. Reports will be generated on a regular basis related to recruitment and data completeness. Components of the reports will be site-specific and include the number of participants screened, number of participants enrolled, number of participants lost to follow-up, key demographics of enrolled participants, number of missing or deficient forms, and summaries of data audits and edits.

12.3 Data Audits:

A list of completed forms for specific participants will be randomly selected from the study database on a regular basis. We will also inspect the forms for proper tracking of modifications and completion of fields not captured in the study database. Discrepancies between the study forms and database and any errors found on the forms will be reported back to the study coordinators and resolution required.

12.4 Data Edits:

The data entry process will be guided by range and logic checks built into the study database. However, the entry of inappropriate data is still possible, so data edits will be run on the study database on a regular basis (approximately quarterly). Site-specific data edit reports will be generated for missing, out-of-range, unusual and inconsistent values. Study staff must respond to each data edit and make modifications to the study form and database as needed.
