# Supplementary material for: Effectiveness of a Multicomponent Intervention to Reduce Multidrug-Resistant Organisms in Nursing Homes: A Cluster Randomized Clinical Trial
Source: JAMA Netw Open. 2021 Jul 16;4(7):e2116555. doi: 10.1001/jamanetworkopen.2021.16555 (PMC8285736; doi:10.1001/jamanetworkopen.2021.16555)
Supplement: Supplement 2. — eTable 1. Infection Prevention Practices of Intervention and Control Group Nursing Homes eTable 2. Facility and Participant Characteristics eFigure 1. Conceptual Model Depicting the Interaction Between Environmental Contamination and Patient MDRO Burden eTable 3. Characteristics of Patients with at Least 1 Follow-Up Visit eTable 4. Microbial Swab-Level Survey of MDROs During a Multicomponent Intervention eFigure 2. Visit-Level Proportion of Patient Colonization and Environmental Contamination by Intervention Status eTable 5. Microbial Survey Results for Individual MDROs on Patients by Intervention Status eTable 6. Microbial Survey Results for Individual MDROs on High-Touch Environmental Surfaces by Intervention Status eTable 7. Onset of New Physician-Documented Infections eTable 8. Impact of the Multicomponent Intervention on Quality of Care eTable 9. Distribution of Patient Activities in the 30 Minutes Preceding the Study Visit by Intervention Status eTable 10. Patient Hand Contamination with Any MDRO by Patient Activities in the 30 Minutes Preceding the Study Visit by Intervention Status [file jamanetwopen-e2116555-s002.pdf]

Supplemental Online Content

Mody L, Gontjes KJ, Cassone M, et al. Effectiveness of a multicomponent intervention to reduce multidrug-resistant organisms in nursing homes: a cluster randomized clinical trial. *JAMA Netw Open.* 2021;4(7):e2116555.
doi:10.1001/jamanetworkopen.2021.16555

eTable 1. Infection Prevention Practices of Intervention and Control Group Nursing Homes
eTable 2. Facility and Participant Characteristics
eFigure 1. Conceptual Model Depicting the Interaction Between Environmental Contamination and Patient MDRO Burden
eTable 3. Characteristics of Patients with at Least 1 Follow-Up Visit
eTable 4. Microbial Swab-Level Survey of MDROs During a Multicomponent Intervention
eFigure 2. Visit-Level Proportion of Patient Colonization and Environmental Contamination by Intervention Status
eTable 5. Microbial Survey Results for Individual MDROs on Patients by Intervention Status
eTable 6. Microbial Survey Results for Individual MDROs on High-Touch Environmental Surfaces by Intervention Status
eTable 7. Onset of New Physician-Documented Infections
eTable 8. Impact of the Multicomponent Intervention on Quality of Care
eTable 9. Distribution of Patient Activities in the 30 Minutes Preceding the Study Visit by Intervention Status
eTable 10. Patient Hand Contamination with Any MDRO by Patient Activities in the 30 Minutes Preceding the Study Visit by Intervention Status

This supplemental material has been provided by the authors to give readers additional information about their work.

© 2021 Mody L et al. *JAMA Network Open.*

eTable 1. Infection Prevention Practices of Intervention and Control Group Nursing Homes

Infection Prevention Program Elements	Intervention Group (N=3)	Control Group (N=3)
**Infection Prevention Staffing Presence**		
No. NHs with a full-time or part-time infection preventionist	3/3 (100.0%)	3/3 (100.0%)
No. NHs where infection preventionists have a written job description	3/3 (100.0%)	3/3 (100.0%)
**Surveillance**		
No. NHs with a surveillance system for MDROs and infections	3/3 (100.0%)	3/3 (100.0%)
No. NHs that record infection rates of the facility/residents	3/3 (100.0%)	3/3 (100.0%)
No. NHs that monitor antibiotic use	3/3 (100.0%)	3/3 (100.0%)
**Standard and Transmission-Based Precautions**		
No. NHs with isolation and precautions policies in place	3/3 (100.0%)	3/3 (100.0%)
**Hand Hygiene**		
No. NHs with a hand hygiene program	3/3 (100.0%)	3/3 (100.0%)
No. NHs with hand sanitizers available for staff use	3/3 (100.0%)	3/3 (100.0%)
No. NHs with alcohol-based gels available for staff use	3/3 (100.0%)	3/3 (100.0%)

Abbreviations: MDRO, multidrug-resistant organisms; NH, nursing homes.

Enrolled NHs were interviewed by trained research personnel about their infection prevention practices and policies prior to study enrollment (either in December 2014 or January 2015). Given the unstructured survey design, questions with missing or uninterpretable responses were excluded. Hence, while similarities in programming were observed, the implementation of these program components and other unmentioned program components may vary across facilities.

© 2021 Mody L et al. *JAMA Network Open.*

eTable 2. Facility and Participant Characteristics

Facility No.	Ownership Status	2016 Star Rating	Beds, No.	Age, y, Mean (SD)	Male, No. (%)	Functional Status[a], Mean (SD)	Comorbidity Score[b], Mean (SD)
**Intervention Group**							
1 (n=43)	For-profit	5	74	78.8 (11.3)	16 (37.2)	13.3 (4.4)	1.9 (1.6)
2 (n=38)	For-profit	3	143	71.4 (11.1)	16 (42.1)	13.6 (4.7)[c]	2.8 (1.9)
6 (n=32)	For-profit	3	102	62.3 (13.8)	16 (50.0)	12.2 (3.4)	2.5 (1.8)
**Total (n=113)[d]**		**3.7 (1.2)**	**106.3 (34.7)**	**71.6 (13.6)**	**48 (42.5)**	**13.1 (4.2)**	**2.4 (1.8)**
**Control Group**							
3 (n=66)	For-profit	4	103	76.6 (11.7)	30 (45.5)	15.0 (4.4)[c]	2.9 (2.0)
4 (n=26)	For-profit	3	88	80.8 (11.5)	13 (50.0)	15.7 (4.4)[c]	2.5 (1.7)
5 (n=40)	For-profit	4	133	62.7 (11.6)	20 (50.0)	11.8 (3.2)	2.1 (1.8)
**Total (n=132)[d]**		**3.7 (0.6)**	**108 (22.9)**	**73.2 (13.6)**	**63 (47.7)**	**14.1 (4.3)**	**2.6 (1.9)**

Abbreviation: SD, standard deviation.

[a] Functional status was assessed using the Physical Self-Maintenance Scale.
[b] Comorbidity score was assessed using the Charlson Comorbidity Index.
[c] Each facility had one missing observation.
[d] Mean (SD) unless otherwise noted.

© 2021 Mody L et al. *JAMA Network Open.*

eFigure 1. Conceptual Model Depicting the Interaction Between Patient MDRO Burden and Environmental Contamination

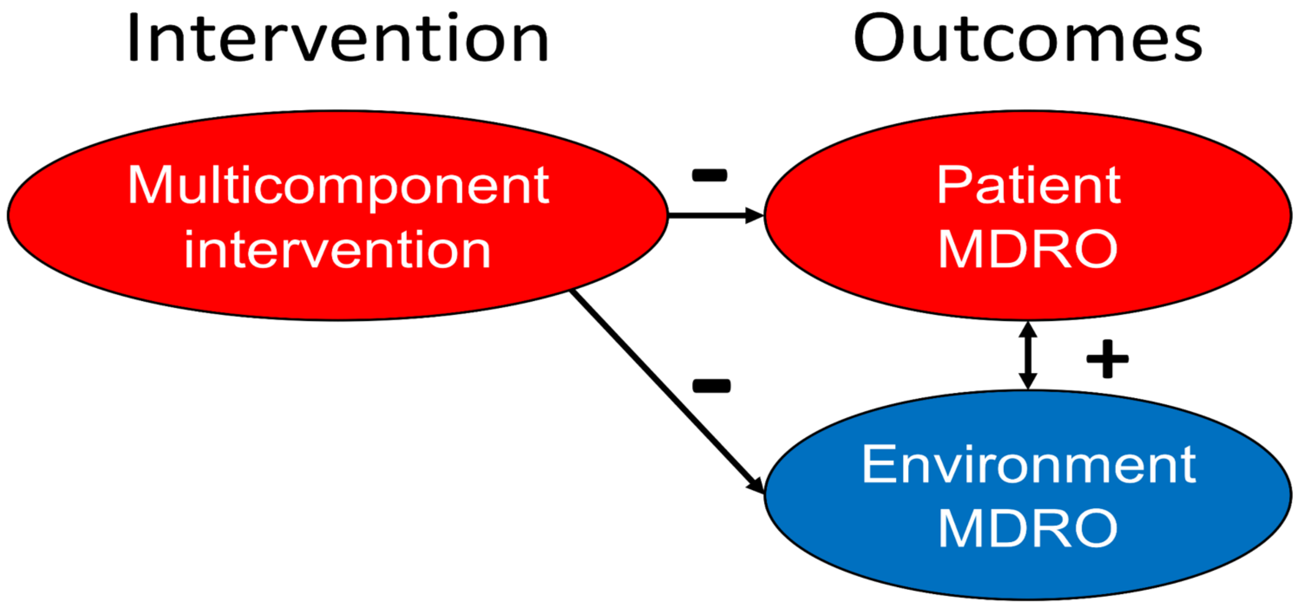

Abbreviation: MDRO, multidrug-resistant organism.

Traditional cluster-randomized clinical trials only capture patient outcomes. In our analytical model, the patient is the actor and the patient's environment can potentially interact with the patient at each visit.

© 2021 Mody L et al. *JAMA Network Open.*

eTable 3. Characteristics of Patients with at Least 1 Follow-Up Visit

Characteristics	Patients With >1 Follow-Up Visit	
	**Intervention Group (n=89)**	**Control Group (n=110)**
Age, y, mean (SD)	72.6 (13.1)	73.5 (13.5)
Male sex, No. (%)	37 (41.6)	51 (46.4)
Race[a]		
Non-Hispanic White, No. (%)	36 (40.5)	73 (67.0)
African American, No. (%)	53 (59.5)	36 (33.0)
Antibiotic use past 30 d, No. (%)[a]	49 (58.3)	58 (56.3)
Charlson Comorbidity Index score, median (IQR)	2 (1-4)	2 (1-3)
Physical Self-Maintenance Scale score, median (IQR)[b]	11.5 (10.0-15.0)	14.0 (11.0-17.5)
Ambulation, No. (%)[a]	39 (43.8)	64 (58.7)
Dressing, No. (%)[a]	35 (39.3)	70 (64.2)
Bathing, No. (%)	34 (38.2)	61 (55.5)
Toileting, No. (%)[a]	24 (27.0)	40 (36.7)
Grooming, No. (%)	17 (19.1)	35 (31.8)
Feeding, No. (%)[a]	3 (3.4)	7 (6.4)
Device use on enrollment, No. (%)	9 (10.1)	19 (17.3)
Feeding tube, No. (%)	2 (2.3)	5 (4.6)
Urinary catheter, No. (%)	7 (7.9)	15 (13.6)
Percutaneously inserted central line, No. (%)	4 (4.5)	5 (4.6)
History of MRSA, No. (%)[a]	3 (3.6)	3 (3.0)
History of VRE, No. (%)[a]	1 (1.2)	1 (1.0)
History of R-GNB, No. (%)[b]	2 (2.4)	3 (3.0)
Open wounds on enrollment, No. (%)[a]	11 (12.5)	16 (14.8)
Admitted from hospital, No. (%)[a]	85 (98.8)	104 (98.1)
Length of preadmission hospitalization > 14 d, No. (%)[a]	11 (12.5)	8 (7.3)
Anticipated short-stay patient, No. (%)[a]	85 (95.5)	102 (93.6)
Days of follow-up, median (IQR)	17 (7-27)	20 (9-28)

Abbreviations: IQR, interquartile range; MRSA, methicillin-resistant *Staphylococcus aureus*; R-GNB, resistant gram-negative bacilli; SD, standard deviation; VRE, vancomycin-resistant enterococci.

[a] Due to data missing on admission, the following total sample sizes apply: race, n=198; antibiotic use in the past 30 days, n=187; physical-self maintenance score, n=196; ambulation, n=198; dressing, n=198; toileting, n=198; feeding, n=198; history of MRSA, n=184; history of VRE, n=187; history of R-GNB, n=182; open wounds on enrollment, n=196; admitted from hospital, n=192; length of preadmission hospitalization > 14 days, n=198; anticipated short-stay patient, n=198.
[b] Functional disabilities are defined as (1) ambulation: ambulates with assistance of another person, uses a wheelchair with help getting in and out, or cannot move without help; (2) dressing: requires at least moderate assistance with dressing; (3) bathing: unable to independently bathe more than hands and face; (4) toileting: soiling or wetting more than once a week; (5) grooming: regularly needs at least moderate assistance or supervision in grooming; (6) feeding: eats with moderate assistance and is untidy.

© 2021 Mody L et al. *JAMA Network Open.*

eTable 4. Microbial Swab-Level Survey of MDROs During a Multicomponent Intervention

Variable	No. Positive Swabs (%)		Unadjusted OR (95% CI)
	Intervention (Pt N=1556, Env N=2372)	Control (Pt N=2098, Env N=3234)	
**Any MDRO**			
Patient Colonization	245 (15.8)	434 (20.7)	0.72 (0.60-0.85)
Environment Contamination	309 (13.0)	555 (17.2)	0.72 (0.62-0.84)
**MRSA**			
Patient Colonization[a]	93 (6.0)	169 (8.1)	0.73 (0.56-0.94)
Environment Contamination[b]	122 (5.2)	232 (7.2)	0.70 (0.56-0.88)
**VRE**			
Patient Colonization	102 (6.6)	208 (9.9)	0.64 (0.50-0.82)
Environment Contamination[c]	152 (6.4)	308 (9.5)	0.65 (0.53-0.80)
**R-GNB**			
Patient Colonization[d]	81 (5.2)	151 (7.2)	0.71 (0.54-0.94)
Environment Contamination	66 (2.8)	106 (3.3)	0.84 (0.62-1.15)

Abbreviations: CI, confidence interval; Env, environment swabs; MDRO, multidrug-resistant organism; MRSA, methicillin-resistant *Staphylococcus aureus*; OR, odds ratio; Pt, patient swabs; R-GNB, resistant Gram-negative bacilli; VRE, vancomycin-resistant enterococci.

Due to missing data, the following total sample sizes apply: [a], n = 1553 and 2098 swabs, respectively; [b], n = 2366 and 3233 swabs, respectively; [c], n = 2371 and 3234 swabs, respectively; [d], n = 1554 and 2096 swabs, respectively.

© 2021 Mody L et al. *JAMA Network Open.*

eFigure 2. Visit-Level Proportion of Patient Colonization and Environmental Contamination by Intervention Status

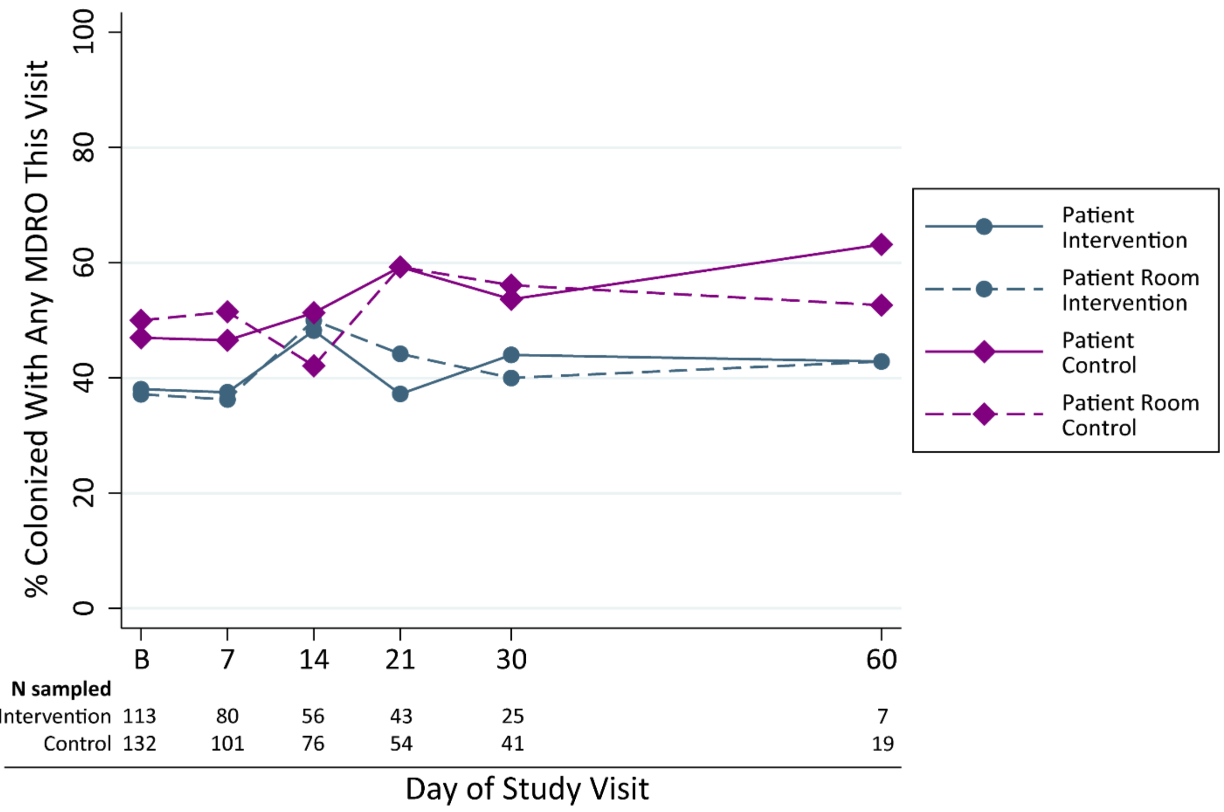

Abbreviation: B, baseline. To demonstrate the different proportion of specimens positive over time, we plot the proportion of specimens positive for each specimen category (patient and room environment) at each study visit, stratified by intervention status. Due to low sample size at the later visits, this figure is restricted up to study visit on day 60.

© 2021 Mody L et al. *JAMA Network Open.*

eTable 5. Microbial Survey Results for Individual MDROs on Patient by Intervention Status

	Number of MDRO-Positive Samples No. Positive Samples/No. Samples Collected (%)		
	**Intervention**	**Control**	**P value[a]**
**Any MDRO**	**245/1556 (15.8)**	**434/2098 (20.7)**	**<0.001**
Nares	34/334 (10.2)	54/449 (12.0)	0.42
Oropharynx	27/327 (8.3)	60/442 (13.6)	0.02
Groin	64/339 (18.9)	101/450 (22.4)	0.22
Perianal Area	46/192 (24.0)	96/267 (36.0)	0.006
Hand	68/339 (20.1)	108/452 (23.9)	0.20
Feeding Tube Site	2/7 (28.6)	2/20 (10.0)	0.27
Suprapubic Catheter Site	3/10 (30.0)	9/13 (69.2)	0.10
Wound	1/8 (12.5)	4/5 (80.0)	0.03
**MRSA**	**93/1553 (6.0)**	**169/2098 (8.1)**	**0.02**
Nares	28/332 (8.4)	48/449 (10.7)	0.29
Oropharynx	14/327 (4.3)	21/442 (4.8)	0.76
Groin	10/339 (3.0)	17/450 (3.8)	0.53
Perianal Area	2/192 (1.0)	15/267 (5.6)	0.01
Hand	37/338 (11.0)	56/452 (12.4)	0.53
Feeding Tube Site	2/7 (28.6)	1/20 (5.0)	0.16
Suprapubic Catheter Site	0/10 (0.0)	7/13 (53.9)	NA
Wound	0/8 (0.0)	4/5 (80.0)	NA
**VRE**	**102/1556 (6.6)**	**208/2098 (9.9)**	**<0.001**
Nares	3/334 (0.90)	3/449 (0.7)	0.70
Oropharynx	11/327 (3.4)	26/442 (5.9)	0.11
Groin	33/339 (9.7)	56/450 (12.4)	0.23
Perianal Area	28/192 (14.6)	62/267 (23.2)	0.02
Hand	26/339 (7.7)	60/452 (13.3)	0.01
Feeding Tube Site	0/7 (0.0)	0/20 (0.0)	NA
Suprapubic Catheter Site	0/10 (0.0)	1/13 (7.7)	NA
Wound	1/8 (12.5)	0/5 (0.0)	NA
**R-GNB**	**81/1554 (5.2)**	**151/2096 (7.2)**	**0.02**
Nares	4/334 (1.2)	4/448 (0.9)	0.73
Oropharynx	5/325 (1.5)	22/442 (5.0)	0.01
Groin	32/339 (9.4)	58/450 (12.9)	0.13
Perianal Area	26/192 (13.5)	48/266 (18.1)	0.20
Hand	11/339 (3.2)	13/452 (2.9)	0.77

© 2021 Mody L et al. *JAMA Network Open.*

	Number of MDRO-Positive Samples<br>No. Positive Samples/No. Samples Collected (%)		
	**Intervention**	**Control**	**P value[a]**
**R-GNB**			
Feeding Tube Site	0/7 (0.0)	1/20 (5.0)	NA
Suprapubic Catheter Site	3/10 (30.0)	4/13 (30.8)	1.00
Wound	0/8 (0.0)	1/5 (20.0)	NA

Abbreviations: MDRO, multidrug-resistant organism; MRSA, methicillin-resistant *Staphylococcus aureus*; R-GNB, resistant-gram negative bacilli; VRE, vancomycin-resistant enterococci.

[a] Swab-level differences were assessed using a chi-squared test or Fisher's exact test.

© 2021 Mody L et al. *JAMA Network Open.*

eTable 6. Microbial Survey Results for Individual MDROs on High-Touch Environmental Surfaces by Intervention Status

	**Number of MDRO-Positive Samples No. Positive Samples/No. Samples Collected (%)**		
	**Intervention**	**Control**	**P value[a]**
**Any MDRO**	**309/2372 (13.0)**	**555/3234 (17.2)**	**<0.001**
Bed Controls	41/336 (12.2)	73/449 (16.3)	0.11
Bedside Table	56/337 (16.6)	91/451 (20.2)	0.20
Call Button	37/331 (11.2)	65/450 (14.4)	0.18
Toilet Seat	52/309 (16.8)	97/399 (24.3)	0.02
Doorknob	20/332 (6.0)	43/448 (9.6)	0.07
TV Remote	53/287 (18.5)	101/401 (25.2)	0.04
Bedrail	9/54 (16.7)	26/135 (19.3)	0.68
Wheelchair	31/270 (11.5)	38/393 (9.7)	0.45
Walker	2/45 (4.4)	3/32 (9.4)	0.64
O2 Pump/Concentrator/Tank	7/53 (13.2)	7/44 (15.9)	0.71
IV Pump	0/11 (0.0)	1/17 (5.9)	NA
Feeding Pump	1/7 (14.3)	10/15 (66.7)	0.06
**MRSA**	**122/2366 (5.2)**	**232/3233 (7.2)**	**0.002**
Bed Controls	14/335 (4.2)	34/449 (7.6)	0.05
Bedside Table	26/336 (7.7)	38/451 (8.4)	0.73
Call Button	14/331 (4.2)	28/450 (6.2)	0.22
Toilet Seat	15/308 (4.9)	28/399 (7.0)	0.24
Doorknob	11/331 (3.3)	17/448 (3.8)	0.73
TV Remote	20/287 (7.0)	44/401 (11.0)	0.08
Bedrail	5/54 (9.3)	20/135 (14.8)	0.31
Wheelchair	12/269 (4.5)	17/392 (4.3)	0.94
Walker	0/45 (0.0)	1/32 (3.1)	0.42
O2 Pump/Concentrator/Tank	4/52 (7.7)	3/44 (6.8)	1.00
IV Pump	0/11 (0.0)	0/17 (0.0)	NA
Feeding Pump	1/7 (14.3)	2/15 (13.3)	1.00
**VRE**	**152/2371 (6.4)**	**308/3234 (9.5)**	**<0.001**
Bed Controls	26/336 (7.7)	41/449 (9.1)	0.49
Bedside Table	24/337 (7.1)	44/451 (9.8)	0.19
Call Button	23/331 (7.0)	41/450 (9.1)	0.28
Toilet Seat	29/309 (9.4)	67/399 (16.8)	0.004
Doorknob	7/331 (2.1)	23/448 (5.1)	0.03

© 2021 Mody L et al. *JAMA Network Open.*

	Number of MDRO-Positive Samples No. Positive Samples/No. Samples Collected (%)		
	Intervention	Control	P value[a]
**VRE**			
TV Remote	25/287 (8.7)	50/401 (12.5)	0.12
Bedrail	4/54 (7.4)	10/135 (7.4)	1.00
Wheelchair	11/270 (4.1)	15/393 (3.8)	0.87
Walker	1/45 (2.2)	2/32 (6.3)	0.57
O2 Pump/Concentrator/Tank	2/53 (3.8)	6/44 (13.6)	0.14
IV Pump	0/11 (0.0)	1/17 (5.9)	NA
Feeding Pump	0/7 (0.0)	8/15 (53.3)	NA
**R-GNB**	**66/2372 (2.8)**	**106/3234 (3.3)**	**0.29**
Bed Controls	5/336 (1.5)	7/449 (1.6)	0.94
Bedside Table	15/337 (4.5)	23/451 (5.1)	0.67
Call Button	3/331 (0.9)	4/450 (0.9)	1.00
Toilet Seat	11/309 (3.6)	30/399 (7.5)	0.03
Doorknob	2/332 (0.6)	9/448 (2.0)	0.13
TV Remote	15/287 (5.2)	20/401 (5.0)	0.89
Bedrail	3/54 (5.6)	2/135 (1.5)	0.14
Wheelchair	10/270 (3.7)	7/393 (1.8)	0.12
Walker	1/45 (2.2)	0/32 (0.0)	1.00
O2 Pump/Concentrator/Tank	1/53 (1.9)	2/44 (4.6)	0.59
IV Pump	0/11 (0.0)	0/17 (0.0)	NA
Feeding Pump	0/7 (0.0)	2/15 (13.3)	NA

Abbreviations: MDRO, multidrug-resistant organism; MRSA, methicillin-resistant *Staphylococcus aureus*; R-GNB, resistant-Gram negative bacilli; VRE, vancomycin-resistant enterococci.

[a] Swab-level differences were assessed using a chi-squared test or Fisher's exact test.

© 2021 Mody L et al. *JAMA Network Open.*

eTable 7. Onset of New Physician-Documented Infections

Infections	No. (%) Intervention Group (n = 89)	No. (%) Control Group (n = 110)	P value[a]
Urinary tract infection	6 (6.7)	6 (5.5)	0.77
Lower respiratory tract infection	5 (5.6)[b]	1 (0.9)	0.09
Upper respiratory tract infection	1 (1.1)	3 (2.7)	0.63
Skin & soft tissue infection	3 (3.4)	4 (3.6)	1.00
Clostridioides difficile infection	0 (0.0)	0 (0.0)	NA
Primary bacteremia	0 (0.0)	0 (0.0)	NA
Conjunctivitis	0 (0.0)	3 (2.7)	0.26
Other	2 (2.3)	2 (1.8)	1.00

[a] Fisher's exact test was used to assess whether significant differences in the onset of new physician-documented infections exist between the intervention group and control group nursing homes.
[b] All patients with lower respiratory tract infections in the intervention group had physician-documented pneumonia.

© 2021 Mody L et al. *JAMA Network Open.*

eTable 8. Impact of the Multicomponent Intervention on Quality of Care

Quality Indicator	Intervention Group			Control Group
	**Low-Risk**	**High-Risk**	**Total**	**Total**
**Progress Notes**				
Lookback Observations, No.	65	36	101	164
Progress Notes, No.	778	434	1212	3020
Progress Notes per Lookback Observation, mean (SD)[a]	12.2 (8.9)	12.1 (8.8)	11.8 (8.8)	11.8 (8.7)
P value[b]	0.33	0.38	0.31	
**Therapy Sessions**				
Lookback Observations, No.	56	19	75	111
Therapy Sessions, No.	277	95	372	904
Therapy Sessions per Lookback Observation, mean (SD)[a,c]	4.9 (0.3)	5.0 (0.0)	4.9 (0.6)	4.9 (0.6)
P value[b]	0.26	0.24	0.19	

Abbreviation: SD, standard deviation.

[a] Residents may be included in the total more than once if they were actively participating during each month of the lookback data collection.
[b] P value compares the average number of quality indicators per lookback observation in intervention facilities to the control facilities using the two-sample t-test.
[c] Residents that were not receiving therapy services (e.g., physical, occupational, speech therapy) were excluded from this analysis.

© 2021 Mody L et al. *JAMA Network Open.*

eTable 9. Distribution of Patient Activities in the 30 Minutes Preceding the Study Visit by Intervention Status

Activity[a]	No. (%)		
	Intervention (n = 208 Activities)	Control (n = 263 Activities)	Total
Leisure in room	143 (68.8)	194 (73.8)	337 (71.6)
Eating in room	28 (13.5)	44 (16.7)	72 (15.3)
Eating in dining room	26 (12.5)	16 (6.1)	42 (8.9)
Therapy	18 (8.7)	21 (8.0)	39 (8.3)
Used restroom	18 (8.7)	16 (6.1)	34 (7.2)
In hallway	9 (4.3)	11 (4.2)	20 (4.3)

[a] Patients could report multiple activities.

© 2021 Mody L et al. *JAMA Network Open.*

eTable 10. Patient Hand Contamination with Any MDRO by Patient Activities in the 30 Minutes Preceding the Study Visit by Intervention Status

**Activity**	**No. (%)**		
	**Intervention (n = 208 Activities)**	**Control (n = 263 Activities)**	**Total**
Leisure in room	26/143 (18.2)	45/194 (23.2)	71/337 (21.1)
Eating in room	3/28 (10.7)	11/44 (25.0)	14/72 (19.4)
Eating in dining room	4/26 (15.4)	4/16 (25.0)	8/42 (19.1)
Therapy	2/18 (11.1)	2/21 (9.5)	4/39 (10.3)
Used restroom	0/18 (0)	5/16 (31.3)	5/34 (14.7)
In hallway	0/9 (0)	2/11 (18.2)	2/20 (10.0)

[a] Patients could report multiple activities.

© 2021 Mody L et al. *JAMA Network Open.*
