# Supplementary material for: Effectiveness of a Multicomponent Intervention to Reduce Multidrug-Resistant Organisms in Nursing Homes: A Cluster Randomized Clinical Trial
Source: JAMA Netw Open. 2021 Jul 16;4(7):e2116555. doi: 10.1001/jamanetworkopen.2021.16555 (PMC8285736; doi:10.1001/jamanetworkopen.2021.16555)
Supplement: Supplement 3. — Data Sharing Statement [file jamanetwopen-e2116555-s003.pdf]

Data Sharing Statement

Mody. Effectiveness of a Multicomponent Intervention to Reduce Multidrug-Resistant Organisms in Nursing Homes. *JAMA Netw Open*. Published July 16, 2021. doi:10.1001/jamanetworkopen.2021.16555

Data
Data available: No

Additional Information
Explanation for why data not available: If accepted, we would consult with the funding agency to determine what data can be shared.
